# Population-Based Risk of Psychiatric Disorders Associated With Recurrent Copy Number Variants

**DOI:** 10.1001/jamapsychiatry.2024.1453

**Published:** 2024-06-26

**Authors:** Morteza Vaez, Simone Montalbano, Xabier Calle Sánchez, Kajsa-Lotta Georgii Hellberg, Saeid Rasekhi Dehkordi, Morten Dybdahl Krebs, Joeri Meijsen, John Shorter, Jonas Bybjerg-Grauholm, Preben B. Mortensen, Anders D. Børglum, David M. Hougaard, Merete Nordentoft, Daniel H. Geschwind, Alfonso Buil, Andrew J. Schork, Dorte Helenius, Armin Raznahan, Wesley K. Thompson, Thomas Werge, Andrés Ingason

**Affiliations:** 1 Institute of Biological Psychiatry, Mental Health Services, Copenhagen University Hospital, Roskilde, Denmark; 2 Lundbeck Foundation Initiative for Integrative Psychiatric Research (iPSYCH), Copenhagen and Aarhus, Denmark; 3 Department of Science and Environment, Roskilde University, Roskilde, Denmark; 4 Center for Neonatal Screening, Department for Congenital Disorders, Statens Serum Institut, Copenhagen, Denmark; 5 National Centre for Register-based Research, Aarhus University, Aarhus, Denmark; 6 Department of Biomedicine – Human Genetics and the iSEQ Center, Aarhus University, Aarhus, Denmark; 7 Center for Genomics and Personalized Medicine, Aarhus, Denmark; 8 Mental Health Center Copenhagen, Copenhagen University Hospital, Copenhagen, Denmark; 9 Department of Neurology, University of California, Los Angeles; 10 Department of Human Genetics, University of California, Los Angeles; 11 Center for Autism Research and Treatment, Semel Institute, David Geffen School of Medicine, University of California, Los Angeles; 12 Center for Human Development, University of California, San Diego; 13 Program in Neurobehavioral Genetics, Semel Institute, David Geffen School of Medicine, University of California, Los Angeles; 14 Lundbeck Foundation Center for GeoGenetics, GLOBE Institute, University of Copenhagen, Copenhagen, Denmark; 15 Neurogenomics Division, Translational Genomics Research Institute (TGEN), Phoenix, Arizona; 16 Section on Developmental Neurogenomics, Human Genetics Branch, National Institute of Mental Health Intramural Research Program, Bethesda, Maryland; 17 Laureate Institute for Brain Research, Tulsa, Oklahoma; 18 Department of Clinical Medicine, University of Copenhagen, Copenhagen, Denmark

## Abstract

**Importance:**

Recurrent copy number variants (rCNVs) have been associated with increased risk of psychiatric disorders in case-control studies, but their population-level impact is unknown.

**Objective:**

To provide unbiased population-based estimates of prevalence and risk associated with psychiatric disorders for rCNVs and to compare risks across outcomes, rCNV dosage type (deletions or duplications), and locus features.

**Design, Setting, and Participants:**

This genetic association study is an analysis of data from the Lundbeck Foundation Initiative for Integrative Psychiatric Research (iPSYCH) case-cohort sample of individuals born in Denmark in 1981-2008 and followed up until 2015, including (1) all individuals (n = 92 531) with a hospital discharge diagnosis of attention-deficit/hyperactivity disorder (ADHD), autism spectrum disorder (ASD), bipolar disorder, major depressive disorder (MDD), or schizophrenia spectrum disorder (SSD) and (2) a subcohort (n = 50 625) randomly drawn from the source population. Data were analyzed from January 2021 to August 2023.

**Exposures:**

Carrier status of deletions and duplications at 27 autosomal rCNV loci was determined from neonatal blood samples genotyped on single-nucleotide variant microarrays.

**Main Outcomes and Measures:**

Population-based rCNV prevalence was estimated with a survey model using finite population correction to account for oversampling of cases. Hazard ratio (HR) estimates and 95% CIs for psychiatric disorders were derived using weighted Cox proportional hazard models. Risks were compared across outcomes, dosage type, and locus features using generalized estimating equation models.

**Results:**

A total of 3547 rCNVs were identified in 64 735 individuals assigned male at birth (53.8%) and 55 512 individuals assigned female at birth (46.2%) whose age at the end of follow-up ranged from 7.0 to 34.7 years (mean, 21.8 years). Most observed increases in rCNV-associated risk for ADHD, ASD, or SSD were moderate, and risk estimates were highly correlated across these disorders. Notable exceptions included high ASD-associated risk observed for Prader-Willi/Angelman syndrome duplications (HR, 20.8; 95% CI, 7.9-55). No rCNV was associated with increased MDD risk. Also, rCNV-associated risk was positively correlated with locus size and gene constraint but not with dosage type. Comparison with published case-control and community-based studies revealed a higher prevalence of deletions and lower associated increase in risk for several rCNVs in iPSYCH2015.

**Conclusions and Relevance:**

This study found that several rCNVs were more prevalent and conferred less risk of psychiatric disorders than estimated previously. Most case-control studies overestimate rCNV-associated risk of psychiatric disorders, likely because of selection bias. In an era where genetics is increasingly being clinically applied, these results highlight the importance of population-based risk estimates for genetics-based predictions.

## Key Points

**Question:**

What is the population-based prevalence and psychiatric risk associated with recurrent copy number variants (rCNVs) and how do they differ across outcomes and rCNVs?

**Findings:**

In a genetic association study of the Danish population born in 1981-2008 and followed up until 2015, most rCNV risk profiles were similar across autism, attention-deficit/hyperactivity disorder, and schizophrenia. The magnitude of risk increase correlated with locus size and gene constraint but did not differ between deletions and duplications.

**Meaning:**

Several rCNVs were more prevalent and conferred less risk of psychiatric disorders than estimated previously, which highlights the importance of population-based estimates for implementing genetics-based prediction in health care.

## Teaser

This genetic association study assesses data from a case-cohort sample of individuals born in Denmark to provide population-based estimates of prevalence and risk associated with psychiatric disorders for recurrent copy number variants.

## Introduction

Recurrent copy number variants (rCNVs) arise through nonallelic homologous recombination mediated by low-copy repeat sequences and are maintained at stable population frequencies through high de novo mutation rates, despite often being associated with substantial pathogenicity and reduced reproductive fitness.^1,2,3^ Around 40 loci in the human genome harbor rCNVs conferring risk of intellectual disability, developmental delay, and congenital malformations.^4^ Many of these have also been associated with increased risk of neuropsychiatric disorders; autism spectrum disorder (ASD),^1^ attention-deficit/hyperactivity disorder (ADHD),^5^ and schizophrenia spectrum disorder (SSD),^6,7,8^ while evidence for bipolar disorder (BPD)^9^ and major depressive disorder (MDD)^10^ is limited.

Most rCNV studies have estimated disease-associated risk from case-control samples, often based on highly selected cases (eg, with long-term illness) and controls (eg, screened for lack of any family history of mental illness). While this design increases power for detecting associations between genetic exposures and disease outcomes, it yields exaggerated estimates of the prevalence of the exposure and the associated risk of the outcome. Studies involving community-based samples (eg, the UK Biobank [UKB]) benefit from larger sample sizes and a multitude of available study outcomes.^10,11^ However, the UKB suffers a “healthy volunteer” bias, which affects association estimates in genetic studies.^12^ Two health care system–based biobank studies from the US estimated population-based rCNV risk for a range of conditions but could not estimate risk for specific psychiatric disorders such as ADHD, ASD, and SSD because of small case samples.^13,14^ This lack of population-based risk estimates for rCNVs compromises implementation of genetically informed clinical care and genetic counselling.

The Lundbeck Foundation Initiative for Integrative Psychiatric Research 2012 (iPSYCH2012) case-cohort sample leverages Danish registers and biobanks with full population coverage to maximize study power for psychiatric disorders within a population-based setting.^15^ Our previous studies of rCNVs at 7 loci in iPSYCH2012 found that in many instances, the population-based prevalence was higher, and associated risk of psychiatric disorders lower, than typically reported in case-control studies.^16,17^

Here, we expand our population-based analyses by (1) systematically considering deletions and duplications across all rCNV loci, including many that have been associated with genomic disorders and clinical phenotypes^11^; (2) comparing the effects on risk across psychiatric outcomes and rCNVs; and (3) doing so with a substantially increased sample size (iPSYCH2015),^18^ affording unprecedented precision and power to characterize population-unbiased rCNV risk profiles with respect to psychiatric outcomes. The study follows the Strengthening the Reporting of Observational Studies in Epidemiology (STROBE) reporting guideline on case-cohort studies.^19,20^

## Methods

### Study Design

The study is based on the iPSYCH2015 case-cohort sample,^18^ which is an update and expansion of iPSYCH2012^15^ and includes 140 116 individuals from 1 657 449 singletons born between May 1, 1981, and December 31, 2008, in Denmark, who were residents in Denmark at 1 year of age and have a mother registered in the Danish Civil Registration System.^21^ The case-cohort includes 2 components: first, the cases were all individuals with a clinical diagnosis of ADHD, ASD, BPD, MDD, or SSD until December 31, 2015, obtained from the Psychiatric Central Research Register.^22^ Second, the cohort was 50 615 individuals randomly drawn from the same source population, corresponding to 3% (see the eMethods in Supplement 1 for details).

In addition to the psychiatric diagnosis groups specifically targeted in the iPSYCH2015 case-cohort design, the same information (date of diagnosis) was obtained through Psychiatric Central Research Register^22^ and the Danish National Patient Registry^23^ for intellectual disability and epilepsy (eMethods in Supplement 1). For this study, the Danish Scientific Ethics Committee has, in accordance with Danish legislation, waived the need for informed consent in biomedical research based on existing biobanks.

### Samples and Genotyping

DNA was extracted from neonatal blood spots retrieved from the Danish Neonatal Screening Biobank (DNSB).^24^ Genotyping of samples is described elsewhere.^15,18^ Extraction of B-allele frequency and probe intensities (through log-R-ratio; LRR) was done with GenomeStudio software (Illumina). Samples with a genotyping call rate less than 95% were excluded.^18^ Access to the data and its use for research purposes was granted by the Danish Scientific Ethics Committee, Danish Health Data Authority, Danish data protection agency, and DNSB Steering Committee.

### CNV Detection and Quality Control

We selected rCNV loci based on CNVs studied in the UKB^11^ that are also listed as recurrent CNVs in ClinGen^25^ with some indication of either haploinsufficiency or triplosensitivity^26^ (eMethods and eTable 1 in Supplement 1). PennCNV^27^ calls from these loci were validated using software developed in-house.^28^ We excluded samples with an LRR-SD of 0.35 or greater , B-allele frequency drift of 0.005 or greater, and/or GC-wave factor of 0.02 or greater (eFigure 1 in Supplement 1). We assessed the quality of visual inspection of calls through intrarater and interrater reliability (eTable 2 in Supplement 1) and tested whether intrarater and interrater reliability and the fraction of calls with undetermined carrier status correlated with LRR-SD and/or locus, array, or dosage type (eTable 3 in Supplement 1). We then performed a series of analyses to verify that sensitivity in rCNV detection (the ability of PennCNV to detect true CNVs) and specificity of visual validation (our ability to correctly classify all PennCNV-detected calls as true or false) was not affected by the level of LRR-SD or by array type (eMethods, eFigure 2, and eTables 4-6 in Supplement 1).

### Statistical Analysis

We calculated population-based rCNV prevalence with 95% CIs using the R survey package, with finite population correction to account for oversampling of cases, and compared deletion-vs-duplication prevalence in iPSYCH2015, as well as iPSYCH2015-vs-UKB prevalence, with a Welch test assuming unequal variance and false discovery rate (FDR) to account for multiple testing (eMethods in Supplement 1). We used sex-stratified, weighted Cox proportional hazard (CPH) models from the R survival package to estimate the rCNV-associated risk of the psychiatric disorders targeted by the case-cohort design, with age at first hospital diagnosis as outcome and rCNV carrier status as exposure (eMethods in Supplement 1). Data were analyzed from January 2021 to August 2023.

To test for differences in rCNV-associated risk between pairs of diagnoses (ASD, ADHD, and SSD) we applied generalized estimating equation (GEE) models using the R glmtoolbox package, accounting for the possibility of being diagnosed with both disorders. Details, including of analyses of rCNV dosage type (deletion [del] vs duplication [dup]) and locus features, also involving use of GEE models, are provided in eMethods in Supplement 1.

As a sensitivity analysis, we performed a logistic regression analysis corresponding to each CPH analysis. Only individuals with the corresponding outcome or from the subcohort were included in each analysis, with the diagnosis as a binary outcome and sex, age at end of follow-up, and genotyping array as covariates (eMethods in Supplement 1).

## Results

We processed PennCNV^27^ calls through the QCtreeCNV pipeline^28^ and further quality control (eMethods in Supplement 1), yielding 3547 verified rCNV calls in 120 247 samples: 79 535 cases and 43 311 cohort samples, including an overlap (cases-in-cohort) of 2599 samples. Sample genotype success rate was 85.8% and did not differ across case, cohort, and cases-in-cohort groups (Fisher exact test, simulated *P* = .12). The sample included 64 735 male (53.8%) and 55 512 female (46.2%) individuals (recorded at birth). Age at end of follow-up ranged from 7.0 to 34.7 years (mean [SD], 21.8 [7.0] years). All participants were born in Denmark, but information on self-declared ethnicity was not available. Nine loci were excluded because of low rCNV prevalence (eTable 1 in Supplement 1).

**Figure 1.**
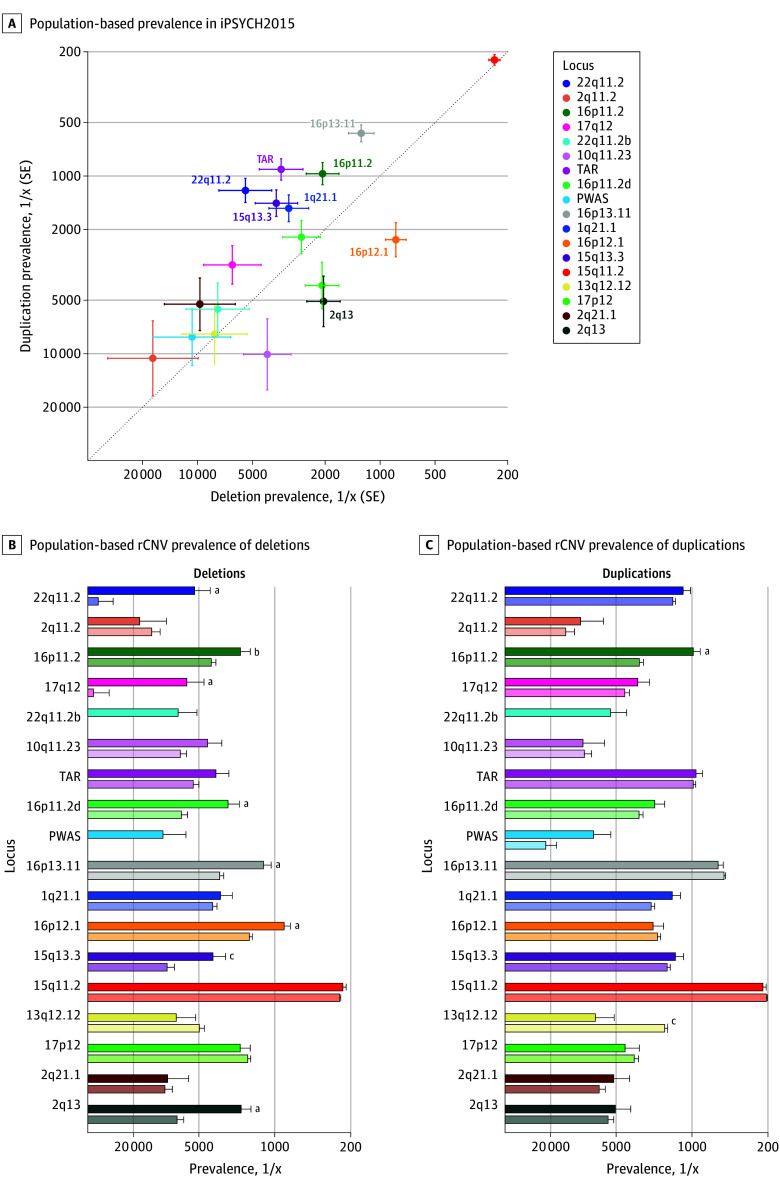
Population-Based Recurrent Copy Number Variant (rCNV) Prevalence in iPSYCH2015 and Comparison With UK Biobank (UKB) A, Population-based prevalence in iPSYCH2015 of deletions and duplications at 18 rCNV loci. Dotted line indicates deletion-duplication prevalence parity. The 8 loci with significant deletion-duplication prevalence disparity (false discovery rate [FDR] *P* < .05) are indicated on the plot. Population-based rCNV prevalence of deletions (B) and duplications (C) in iPSYCH2015 (full color, top) are compared with that reported for the UKB^11^ (semitransparent color, bottom), with loci ordered from top to bottom by decreasing sum loss-of-function observed/expected upper-bound fraction score (eTable 1 in Supplement 1).^29^ In the UKB study, 22q11.2b (turquoise) was not included as an independent locus and the prevalence of PWAS deletion was less than 1 in 100 000.^11^ Error bars indicate the SE of the weighted population-based prevalence in iPSYCH2015 and observed prevalence in UKB, respectively. For detailed results, see eTable 7 in Supplement 1. ^a^FDR *P* < .001. ^b^FDR *P* < .05. ^c^FDR *P* < .01.

Consistent with previous studies,^4,11^ the overall prevalence of duplications was higher than that of deletions (1.29% vs 0.99%, respectively; *P* = 2.2 × 10^−5^). However, the prevalence disparity differed substantially across loci (Figure 1A), with 6 and 2 loci showing significantly higher prevalence of duplications and deletions, respectively (eTable 7 in Supplement 1). When comparing rCNV prevalence with the UKB^11^ for the 17 loci with estimates in both studies, the overall population-based prevalence of deletions was higher in iPSYCH2015 (0.98% vs 0.70%, respectively; *P* = 7.7 × 10^−12^) whereas overall duplication prevalence did not differ significantly (1.27% vs 1.23%, respectively; *P* = .42). In the per-locus comparison, 9 deletions and 1 duplication had higher prevalence (FDR *P* < .05) in iPSYCH2015, 1 duplication was more prevalent in UKB, and 23 rCNVs showed no significant differences (Figure 1B and eTable 7 in Supplement 1).

**Figure 2.**
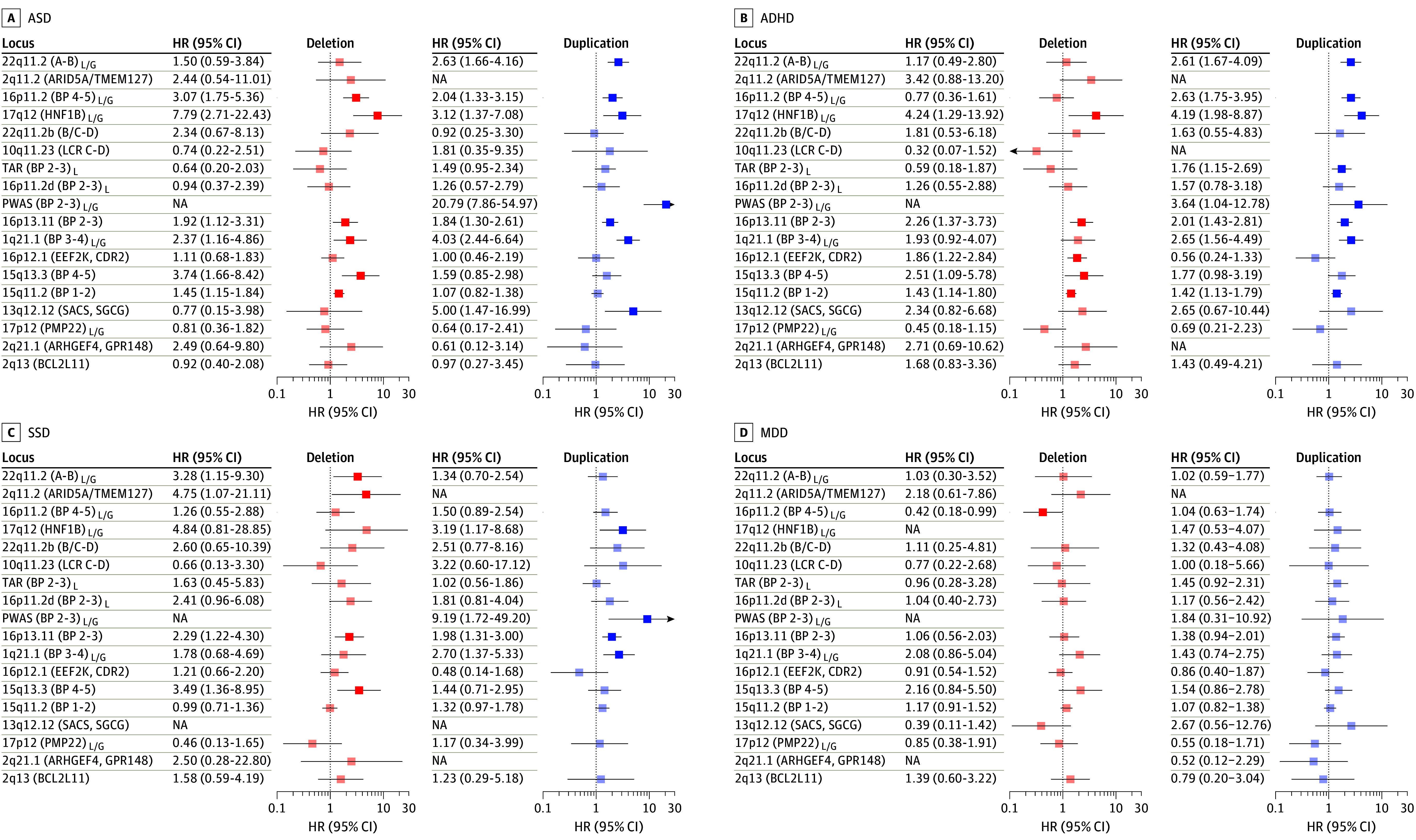
Recurrent Copy Number Variant (rCNV)–Associated Risk of Psychiatric Disorders in the iPSYCH2105 Case Cohort rCNV-associated hazard ratios (HRs) and 95% CIs were derived from Cox proportional hazards (CPH) models using inverse probability of sampling weights and are indicated for deletions and duplications by red and blue, respectively. Loci are ordered on the y-axis by decreasing sum loss-of-function observed/expected upper-bound fraction score.^29^ Locus names include information on associated common breakpoints and/or prominent encompassed genes and indication of curated loss (L) and/or gain (G) disease (eTable 1 in Supplement 1). Comparisons involving less than 2 case carriers, or where the CPH model failed a test of proportionality of hazards, were excluded. NA indicates not assessed.

Per-diagnosis results for the main diagnosis groups (ASD, ADHD, MDD, and SSD) are shown in Figure 2, while a per-locus version of all estimates, including for schizophrenia (*International Statistical Classification of Diseases and Related Health Problems, Tenth Revision,* code F20), BPD, and the broader diagnosis groups (any affective disorder and any iPSYCH2015 disorder) are provided in eFigure 3 in Supplement 1. Risk estimates exceeded 1.5 for a majority of rCNVs tested for ASD (18/34), ADHD (21/32), and SSD (19/31) but only for 6 of 32 tested for MDD (Figure 2). In fact, we observed no significantly increased HRs for MDD (Figure 2) or the broader category of any affective disorder (eTable 8 in Supplement 1). The greatest risk increase was observed for Prader-Willi/Angelman Syndrome (PWAS)-dup with ASD (HR, 20.7; 95% CI, 7.8-54.9) and SSD (HR, 9.1; 95% CI, 1.7-49.2) and for 17q12-del with ADHD (HR, 4.2; 95% CI, 1.2-13.9) (Figure 2).

We compared risk estimates for the main diagnosis groups with the largest available published case-control studies. The number of rCNVs with available estimates in those studies ranged from 11 for ASD^1^ to 13 for ADHD,^5^ 20 for MDD,^10^ and 22 for SSD.^6,7,8^ Risk estimates were significantly lower in our study for 1q21.1-del, 15q11.2-del, 16p11.2-dup, 17p12-del, and 22q11.2-del for SSD; 16p11.2-del and 16p11.2-dup for ASD; and 22q11.2-del for ADHD (eFigure 4 and eTable 9 in Supplement 1). The only significantly higher risk estimate in iPSYCH2015 was for 22q11.2-dup in SSD, for which we find no indication of the protective effect reported in the comparison study.^6^ Our sensitivity analysis found very high correlation across all diagnosis groups between the CPH-derived HRs and corresponding odds ratios (ORs) derived from logistic regression models of iPSYCH2015 data (Pearson correlation test: *r* = 0.94; *P* < 2.2 × 10^−16^) (eTable 10 in Supplement 2).

As no individual rCNV was significantly associated with increased risk of MDD, we assessed the omnibus effect of rCNV carrier status on MDD diagnosis and found no effect of rCNV carrier status on MDD prediction (χ^2^ = 40.32; *P* = .28); a stark contrast to corresponding results for ADHD, ASD, and SSD (χ^2^ > 104.68 and *P* < 1.27 × 10^−8^ for each). Therefore, we excluded MDD from the following comparative analyses of rCNV-associated risk across diagnosis groups, between deletions and duplications, and across loci.

**Figure 3.**
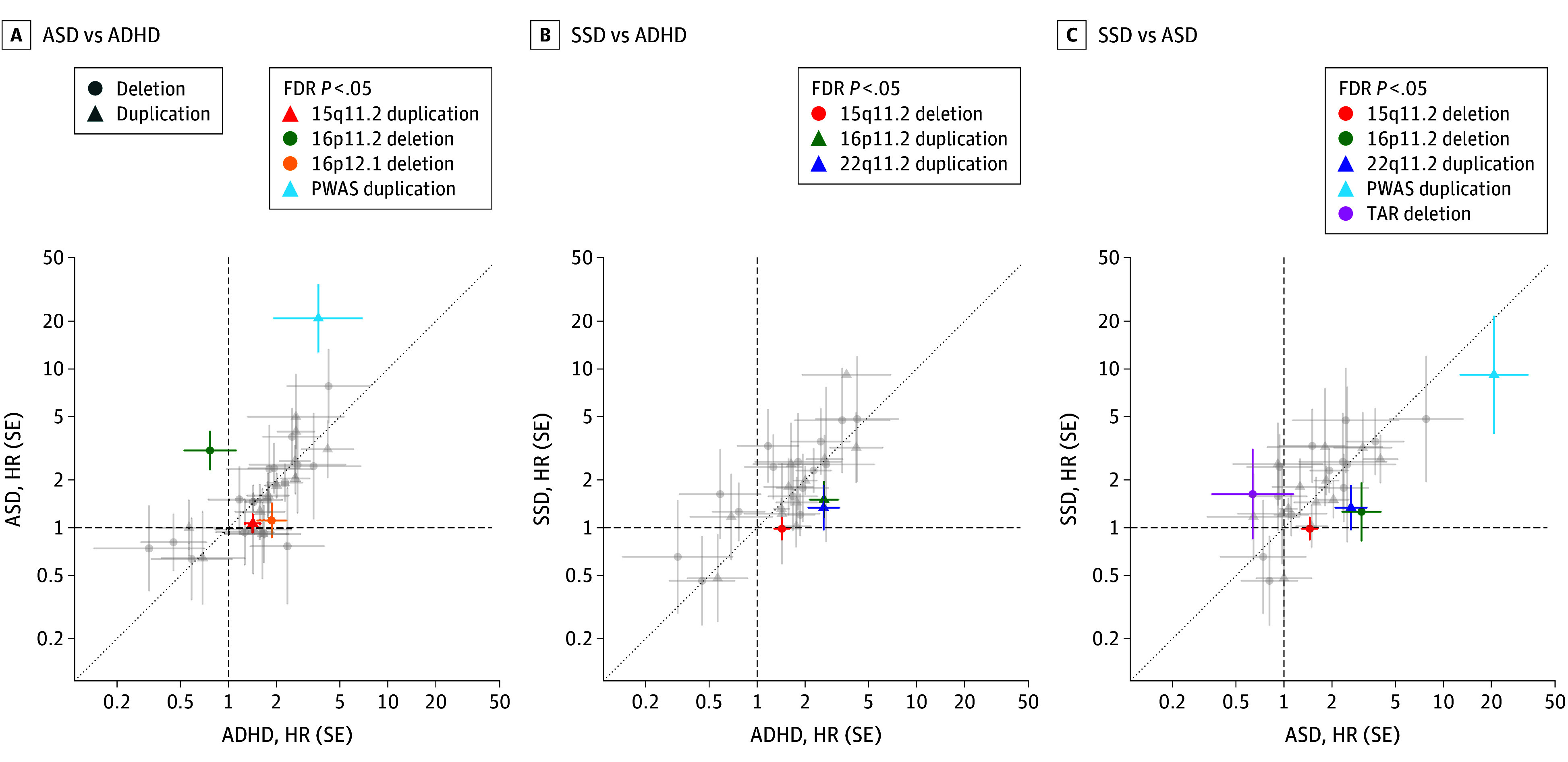
Contrasts in Associated Risk Between Main Psychiatric Outcomes Across Recurrent Copy Number Variants (rCNVs) Hazard ratios (HRs) with error bars indicating SE for rCNV deletions (circles) and duplications (triangles) are plotted in a pairwise comparison for attention-deficit/hyperactivity disorder (ADHD) vs autism spectrum disorder (ASD), schizophrenia spectrum disorder (SSD) vs ASD, and SSD vs ADHD. The dashed line indicates risk parity. On each pairwise comparison plot, we have highlighted those rCNVs showing significant evidence of an rCNV-by-diagnosis interaction in generalized estimating equations predicting case status for 2 diagnoses at a time (false discovery rate [FDR] *P* < .05), with colors corresponding to the rCNV locus in Figure 1.

We tested if rCNV-associated risk varied across outcomes through complementary approaches made possible by having information on multiple outcomes in the same population-based sample. First, we plotted CNV-associated HR estimates for each pair of the remaining diagnosis groups (ADHD, ASD, and SSD) and observed high pairwise correlations (*r*[ASD-ADHD] = 0.68, *P* = 2 × 10^−5^; *r*[ASD-SSD] = 0.71, *P* = 7 × 10^−6^; *r*[ADHD-SSD] = 0.75, *P* = 2 × 10^−6^) (Figure 3). Then, we fitted nested GEE models with and without an interaction term between the diagnosis group and overall rCNV carrier status. This revealed a significant overall difference in rCNV-associated effects between ASD and SSD (χ^2^ = 7.47, *P* = .006), and ADHD and SSD (χ^2^ = 10.08, *P* = .002), but not between ASD and ADHD (χ^2^ = 0.21, *P* = .64). Finally, we used the same approach but replaced the overall rCNV carrier variable with a categorical rCNV variable. This analysis showed a significant effect of adding the rCNV-diagnosis interaction term in all 3 comparisons, suggesting that for each pair of disorders at least some rCNVs differ in effect size (χ^2^[ASD-ADHD] = 94.6, *P* = 4.2 × 10^−8^; χ^2^[ASD-SSD] = 72.9, *P* = 3.1 × 10^−5^; χ^2^[ADHD-SSD] = 50.1, *P* = .01). Post hoc analyses of the interaction coefficients from the GEE analyses revealed no consistent trend for rCNVs conferring higher or lower risk of any of the 3 diagnoses compared with either of the other 2 (eTable 11 and eFigure 5 in Supplement 1), but among rCNVs with significant interaction coefficients (FDR *P* < .05) HRs for both PWAS-dup and 16p11.2-del were higher for ASD than for ADHD or SSD, and HRs for 15q11.2-del and 22q11.2-dup were higher for ASD and ADHD than SSD (Figure 3).

**Figure 4.**
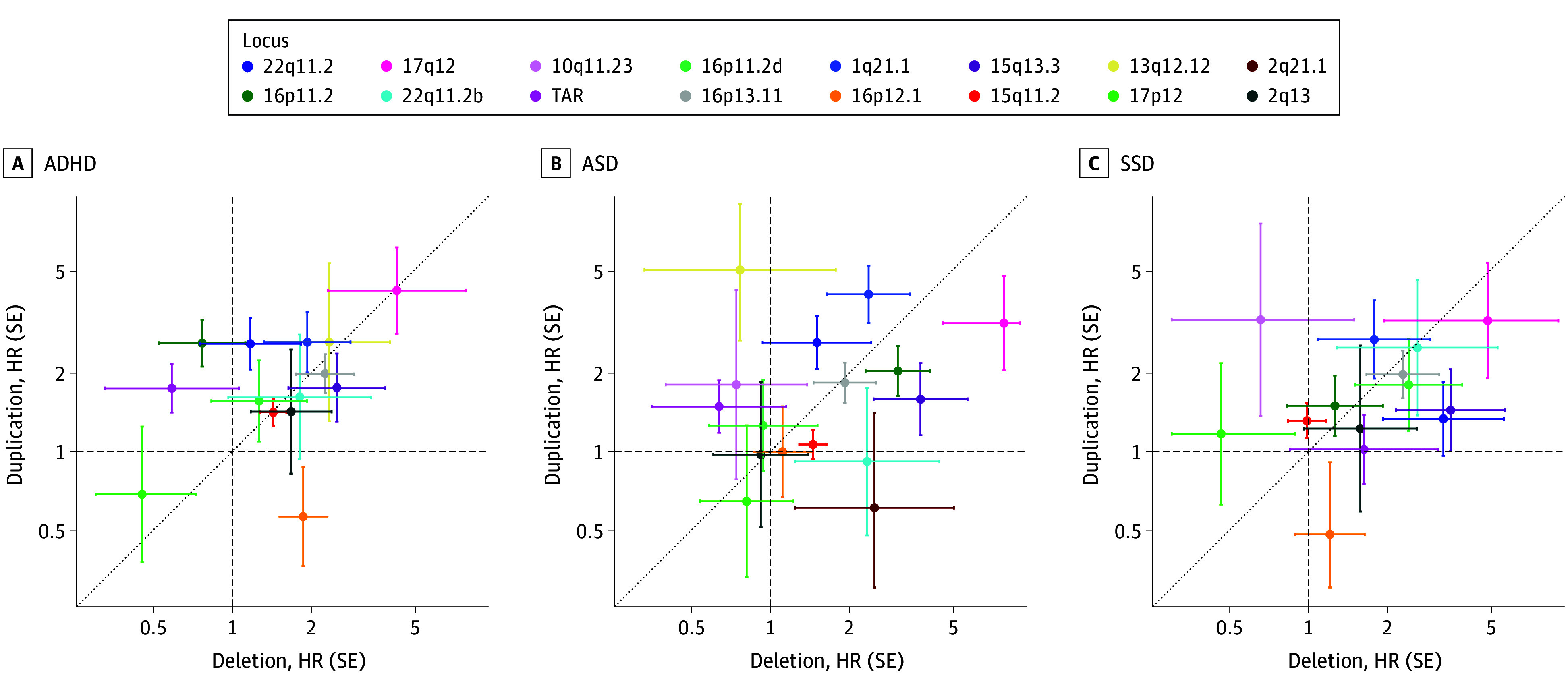
Contrasts in Associated Risk by Dosage Change Across Recurrent Copy Number Variant (rCNV) Loci Hazard ratios (HRs) with error bars indicating SE for deletions and duplications are plotted on the x-axis and y-axis, respectively, for attention-deficit/hyperactivity disorder (ADHD), autism spectrum disorder (ASD), and schizophrenia spectrum disorder (SSD). The 16 loci with available HR estimates for both deletions and duplications for at least 1 diagnosis are indicated by different colors corresponding to previous figures. The dashed line indicates risk parity. We found no evidence of a locus-by-dosage interaction increasing prediction of case status in an overall test across all loci and diagnoses between a generalized estimating equation model with the interaction term and a nested model without the interaction term (likelihood ratio test, *P* = .16).

The distribution of HR estimates for deletions vs duplications across loci for the 3 main diagnosis groups revealed no indication of a consistent dosage-dependent effect (Figure 4), and we found no evidence of an omnibus effect of dosage type on rCNV-associated risk in a formal test across all 3 diagnosis groups (χ^2^ = 0.11, *P* = .73). Also, a comparison of the GEE models with and without an interaction term between rCNV dosage type and locus found no indication of significant locus-specific rCNV dosage type effects (χ^2^ = 20.66, *P* = .14).

**Figure 5.**
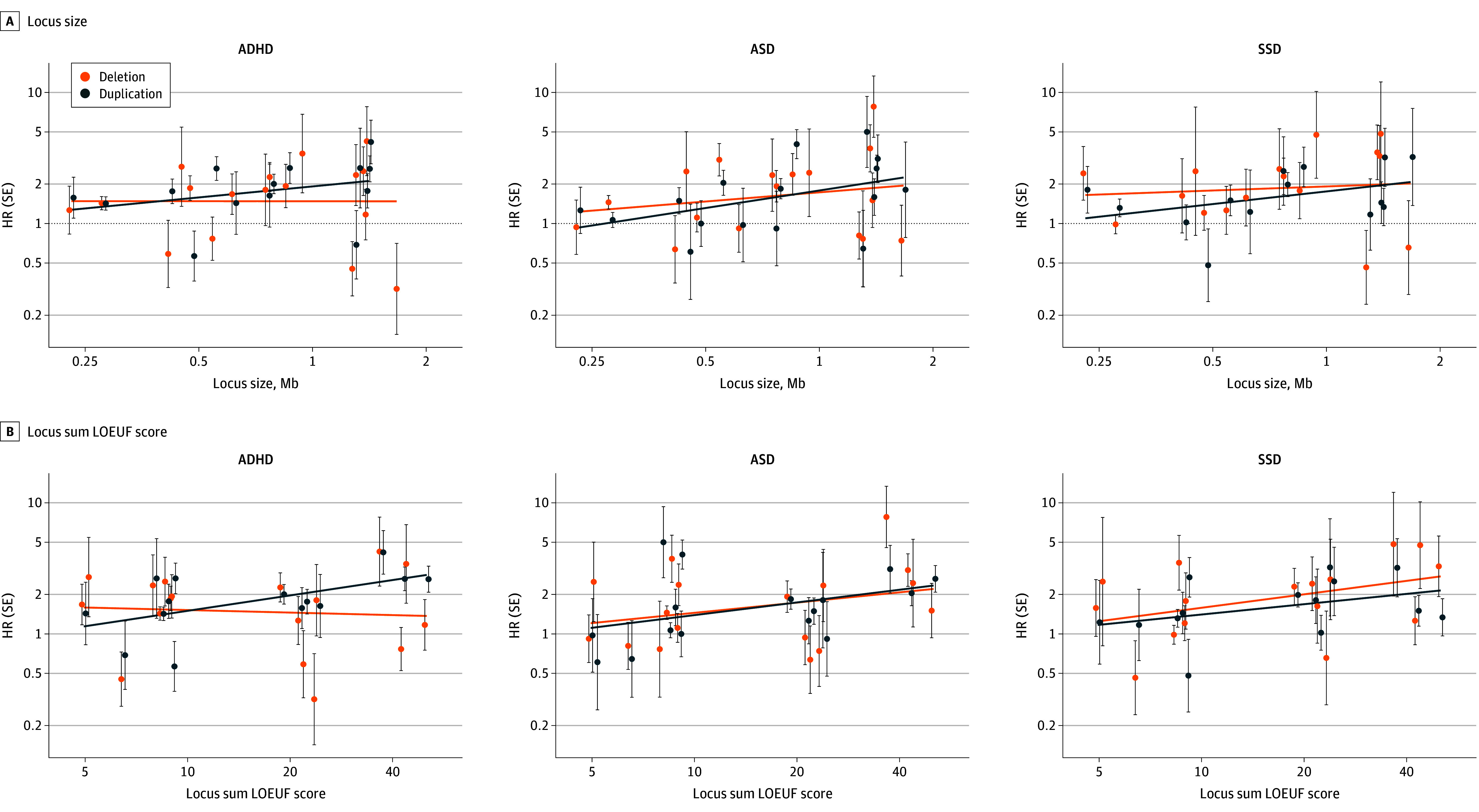
Recurrent Copy Number Variant (rCNV)–Associated Risk as a Function of Locus Properties and rCNV Prevalence Hazard ratios (HR) with error bars indicating SE for deletions and duplications associated with attention-deficit/hyperactivity disorder (ADHD), autism spectrum disorder (ASD), and schizophrenia spectrum disorder (SSD) are plotted against (A) locus size and (B) sum loss-of-function observed/expected upper-bound fraction (LOEUF) score (eTable 1 in Supplement 1 and eTable 12 in Supplement 2).^29^ PWAS duplication was excluded because of outlying locus and effect size. The plots include fitted trend lines. The overall trend for both locus features across all 3 psychiatric outcomes was assessed in a single generalized estimating equation model including all rCNV carriers, and significant positive associations were found for both locus size (*P* = .003) and sum LOEUF score (*P* = .003).

Hypothesizing that rCNV-associated risk correlated with gene content or locus size, we plotted estimated HRs for the 3 disorders against locus size and locus-wide loss-of-function observed/expected upper-bound fraction (LOEUF) score^29^ across loci (Figure 5 in Supplement 1 and eTable 12 in Supplement 2). We also hypothesized that rCNV-associated risk correlated with their population-based prevalence or the difference in prevalence between iPSYCH2015 and UKB^11^ and plotted rCNV prevalence and the iPSYCH2015/UKB prevalence ratio (eFigure 6 in Supplement 1) by fitting log-linear trend lines for deletion- and duplication-associated estimates separately (excluding PWAS because of outlying locus size and HRs). While differing somewhat across diagnoses, the plots indicate that HRs tend to increase with locus size (Figure 5A), LOEUF score (Figure 5B), and iPSYCH/UKB rCNV prevalence ratio (eFigure 6B in Supplement 1), while decreasing with rCNV prevalence itself (eFigure 6A in Supplement 1). A formal test of these trends with nested GEE models confirmed that both locus size and LOEUF score were significantly associated with overall case prediction (size *β*_=_ = 0.09, *P* = .01; LOEUF *β* = 0.10, *P* = .002) as was the iPSYCH/UKB rCNV prevalence ratio (*β* = 0.12, *P* = .009), whereas iPSYCH2015 prevalence was not (*β* = −0.02, *P* = .31).

We applied weighted CPH models to estimate the risk of epilepsy and intellectual disability (ID) associated with rCNVs in our dataset (eMethods in Supplement 1). These disorders were not targeted specifically in the iPSYCH2015 case ascertainment, and the risk estimates are therefore not representative of the source population. Nine rCNVs were significantly associated with increased risk of both epilepsy and ID (out of 28 and 33 tested, respectively), and a further 10 rCNVs were associated with increased risk of one or the other disorder (eFigure 7 and eTable 13 in Supplement 1).

## Discussion

Our assessment of rCNV-associated risk across common psychiatric disorders extends beyond current knowledge because of 2 features of the study design. First, the systematic assessment of deletions and duplications across most known rCNV loci enabled us to fill a gap in the existing literature, especially for ADHD and ASD where our samples are larger and involve more rCNVs than earlier studies.^1,5^ Second, the unbiased population-based study design^18^ allowed critical revising of estimates of rCNV prevalence and associated risk of psychiatric disorders.

iPSYCH2015 includes 5 times as many ASD cases and 3 times as many ADHD cases as the largest published rCNV case-control studies,^1,5^ and risk estimates for a majority of the examined rCNV are novel for both disorders. We observed 6 and 7 rCNVs associated with increased risk of ASD and ADHD, respectively, that had either not been included or not found significant in previous studies. For ASD, we confirmed high risk associated with PWAS-dup and 17q12-del, while 16p11.2 (del and dup) were associated with less increased risk than earlier reported.^1^ For ADHD-associated risk, increases were modest, with HRs ranging from 1.4 at 15q11.2 to 4.2 at 17q12.

While our SSD analysis includes fewer cases than published case-control meta-analyses,^6,7,8^ we provide risk estimates for 9 rCNVs not included in those studies. We confirm high risk associated with PWAS-dup and moderate risk (HRs, 2.0-4.8) associated with 7 other rCNVs. For 6 rCNVs, our estimates differed significantly from previous studies,^6,7,8^ most drastically so for 22q11.2-del (previously reported with OR >50^6^ but with an associated HR of only 3.3 in iPSYCH2015) and 22q11.2-dup (previously reported with strong protective effect against schizophrenia^6^ but not significantly affecting SSD risk in iPSYCH2015).

The comparison of rCNV risk profiles across ASD, ADHD, and SSD suggests that PWAS-dup and 16p11.2-del are associated with a greater increase in risk of ASD than of ADHD or SSD and that 15q11.2-del and 22q11.2-dup are associated with less (if at all any) increase in risk of SSD than of ADHD or ASD. Notwithstanding these interesting signs of variability in risk across diagnosis groups, we observed high correlation of risk estimates for rCNVs across these disorders.

The main exception from this pleiotropic trend was MDD, for which we found no evidence of an overall rCNV-associated increase in risk. This contrasts with the results of 2 US health care system population studies, where overall rCNV carriage associated with a significant increase (OR = 1.5) of depression and/or anxiety risk^13,14^ A limitation to such comparison of overall rCNV effect on risk is that we were unable to include some of the rarest (and potentially most pathogenic) rCNVs that we originally set out to test. A study of the UKB also reported modest increases in MDD risk for a few rCNVs.^10^ The BPD case sample in our study is small and does not add meaningfully to the limited evidence for rCNV involvement in BPD.^9^

Although meiotic nonallelic homologous recombination produces de novo rCNV deletions more often than duplications,^30^ rCNV studies consistently report higher population prevalence of duplications.^4,11^ The general view is that this prevalence disparity reflects a greater effect on viability and reproductive fitness associated with deletions than duplications, a part of which has been attributed to psychiatric disorders. However, we do not observe greater risk associated with deletions than duplications for ASD, ADHD, or SSD. Across the 17 rCNV loci included in our analysis, both locus size and locus-wide LOEUF scores were independently associated with increased disease risk while rCNV dosage type was not. This is consistent with a recent study that found the summed constraint of overlapped genes to best explain CNV-associated risk with ASD.^31^

### Limitations

While the case-cohort design of iPSYCH2015 is optimal to assess rCNV-associated risk of its targeted psychiatric disorders, the relatively young age of participants limited the study power for late-onset psychiatric illness, such as BPD, and the quality of the probe intensity data used to infer CNV carrier status was not as high in the dried blood spot samples used in our study as when using whole blood samples. Also, because of low prevalence, we could not derive estimates for rCNVs at 9 of 27 loci, including some with high reported risk of psychiatric disorders in previous studies (such as 3q29-del and WBS-dup). Third, while having nationwide coverage of hospital-based diagnoses, iPSYCH2015 does not include individuals diagnosed and treated solely outside the hospital regimen, and we additionally note that the diagnostic criteria for specific disorders in some instances differ between iPSYCH2015 and the respective previously published case-control study that we draw comparison to. Finally, iPSYCH2015 was designed to address population-valid risk associated with its targeted common psychiatric disorders, and our observations of less increased risk of these disorders associated with, eg, 22q11.2-del, do not mean that the same is necessarily true of other disorders, such as ID. In fact, from hospital records on ID and epilepsy in our study, we find evidence of high risk of both disorders associated with 22q11.2-del.

## Conclusions

This study found that several rCNVs were more prevalent and conferred less risk of psychiatric disorders than estimated previously. To summarize, we propose that (1) overall, rCNVs are associated with increased risk of psychiatric disorders to a degree that varies according to locus size and gene content but not according to gene dosage; (2) the risk increase is overall similar across ADHD, ASD, and SSD but much less (if at all) pronounced for MDD; (3) some rCNVs, such as PWAS-dup, may not adhere to these general statements; and (4) case-control studies have in some instances overestimated rCNV-associated risk of psychiatric disorders due to selection bias and screening of controls. In an era where genetics is increasingly being used in clinical practice, our results highlight the importance of accurate population-based risk estimates for genetic exposures such as rCNVs.

## Notes

Supplement 1. **eMethods.** Detailed methods description **eFigure 1.** Per-sample CNV-calling quality measures by genotyping array **eFigure 2.** rCNV prevalence difference with UKB vs initial PennCNV specificity **eFigure 3.** rCNVs and risk of psychiatric disorders in iPSYCH2015 (by locus) **eFigure 4.** Comparison of risk estimates between iPSYCH2015 and case-control studies **eFigure 5.** Coefficients of contrasts in rCNV-associated risk between iPSYCH disorders **eFigure 6.** rCNV-associated risk by prevalence and by iPSYCH/UKB prevalence ratio **eFigure 7.** rCNVs and risk of other brain disorders in iPSYCH2015 **eTable 1.** rCNV loci selected for study in the iPSYCH2015 case-cohort sample **eTable 2.** Results of visual inspection of PennCNV calls across included rCNV loci **eTable 3.** Factors affecting initial evaluation of rCNV calls **eTable 4.** Prevalence of rCNVs by genotyping array and LRR-SD **eTable 5.** LRR-SD, case- and QC- fractions, and rCNV prevalence by genotyping wave **eTable 6.** iPSYCH2015 samples by diagnosis group and genotyping array **eTable 7.** rCNV prevalence in iPSYCH2015 and the UKB **eTable 8.** rCNV-associated risk of psychiatric disorders in iPSYCH2015 **eTable 9.** Comparison of risk estimates in iPSYCH2015 with case-control studies **eTable 11.** Diagnosis-specific rCNV effects on risk between iPSYCH2015 disorders **eTable 13.** rCNV-associated risk of other brain disorders in iPSYCH2015
eReferences

Supplement 2. **eTable 10.** Sensitivity analysis of rCNV-associated risk of iPSYCH2015 outcomes **eTable 12.** Gene constraint at rCNV loci

Supplement 3. Nonauthor collaborators

Supplement 4. Data sharing statement
