# Supplementary material for: Population-Based Risk of Psychiatric Disorders Associated With Recurrent Copy Number Variants
Source: JAMA Psychiatry. 2024 Jun 26;81(10):957–66. doi: 10.1001/jamapsychiatry.2024.1453 (PMC11209205; doi:10.1001/jamapsychiatry.2024.1453)
Supplement: Supplement 1. — eMethods. Detailed methods description eFigure 1. Per-sample CNV-calling quality measures by genotyping array eFigure 2. rCNV prevalence difference with UKB vs initial PennCNV specificity eFigure 3. rCNVs and risk of psychiatric disorders in iPSYCH2015 (by locus) eFigure 4. Comparison of risk estimates between iPSYCH2015 and case-control studies eFigure 5. Coefficients of contrasts in rCNV-associated risk between iPSYCH disorders eFigure 6. rCNV-associated risk by prevalence and by iPSYCH/UKB prevalence ratio eFigure 7. rCNVs and risk of other brain disorders in iPSYCH2015 eTable 1. rCNV loci selected for study in the iPSYCH2015 case-cohort sample eTable 2. Results of visual inspection of PennCNV calls across included rCNV loci eTable 3. Factors affecting initial evaluation of rCNV calls eTable 4. Prevalence of rCNVs by genotyping array and LRR-SD eTable 5. LRR-SD, case- and QC- fractions, and rCNV prevalence by genotyping wave eTable 6. iPSYCH2015 samples by diagnosis group and genotyping array eTable 7. rCNV prevalence in iPSYCH2015 and the UKB eTable 8. rCNV-associated risk of psychiatric disorders in iPSYCH2015 eTable 9. Comparison of risk estimates in iPSYCH2015 with case-control studies eTable 11. Diagnosis-specific rCNV effects on risk between iPSYCH2015 disorders eTable 13. rCNV-associated risk of other brain disorders in iPSYCH2015 eReferences [file jamapsychiatry-e241453-s001.pdf]

Supplemental Online Content

Vaez M, Montalbano S, Calle Sánchez X, et al; iPSYCH Investigators. Population-based risk of psychiatric disorders associated with recurrent copy number variants. *JAMA Psychiatry*. Published online June 26, 2024. doi:10.1001/jamapsychiatry.2024.1453

eMethods. Detailed methods description
eFigure 1. Per-sample CNV-calling quality measures by genotyping array
eFigure 2. rCNV prevalence difference with UKB vs initial PennCNV specificity
eFigure 3. rCNVs and risk of psychiatric disorders in iPSYCH2015 (by locus)
eFigure 4. Comparison of risk estimates between iPSYCH2015 and case-control studies
eFigure 5. Coefficients of contrasts in rCNV-associated risk between iPSYCH disorders
eFigure 6. rCNV-associated risk by prevalence and by iPSYCH/UKB prevalence ratio
eFigure 7. rCNVs and risk of other brain disorders in iPSYCH2015
eTable 1. rCNV loci selected for study in the iPSYCH2015 case-cohort sample
eTable 2. Results of visual inspection of PennCNV calls across included rCNV loci
eTable 3. Factors affecting initial evaluation of rCNV calls
eTable 4. Prevalence of rCNVs by genotyping array and LRR-SD
eTable 5. LRR-SD, case- and QC- fractions, and rCNV prevalence by genotyping wave
eTable 6. iPSYCH2015 samples by diagnosis group and genotyping array
eTable 7. rCNV prevalence in iPSYCH2015 and the UKB
eTable 8. rCNV-associated risk of psychiatric disorders in iPSYCH2015
eTable 9. Comparison of risk estimates in iPSYCH2015 with case-control studies
eTable 10. Sensitivity analysis of rCNV-associated risk of iPSYCH2015 outcomes
eTable 11. Diagnosis-specific rCNV effects on risk between iPSYCH2015 disorders
eTable 12. Gene constraint at rCNV loci
eTable 13. rCNV-associated risk of other brain disorders in iPSYCH2015
eReferences

This supplemental material has been provided by the authors to give readers additional information about their work.

eMethods: Detailed methods description

Study design

The current study is based on the iPSYCH2015[1] case-cohort of 140,116 individuals from the 1,657,449 singletons born between May 1, 1981 and December 31, 2008, in Denmark, who were residents in Denmark at 1 year of age and have a mother registered in the Danish Civil Registration System[2]. The case-cohort is made up of two components: (1) Cases: All individuals (n=92,531) who have been clinically diagnosed with major depressive disorder (MDD; ICD10: F32-F33, and ICD-8 296.09, 296.29, 298.09, 300.49, n=37,555), affective disorder (AFF; ICD10: F30-F39, n=40,482), autism spectrum disorder (ASD; ICD10: F84, n=24,975), bipolar disorder (BPD; ICD10: F30-F31, n=3,819), schizophrenia spectrum disorder (SSD; ICD10: F20-F29, n=16,008), schizophrenia (SCZ; ICD10: F20, n=8,113) or attention-deficit/hyperactivity disorder (ADHD; ICD10: F90, n=29,668); according to inpatient and outpatient discharge diagnoses from all Danish hospitals until December 31, 2015, obtained from the Psychiatric Central Research Register (PCRR).[3] (2) Population comparison Cohort: 50,615 individuals randomly drawn from the same birth cohort as the cases, corresponding to roughly 3% of the entire population in Denmark born in 1981-2008. The total number of cases is less than the sum of the subtotal for each diagnosis, as some individuals have more than one diagnosis; and the total number of samples in the study is smaller than the sum of cases and cohort, as the cohort is a random sample of the population and thus includes a small number of cases. In addition to the psychiatric diagnosis groups specifically targeted in the iPSYCH2015 case-cohort design, the same information (date of diagnosis) was obtained through PCRR[3] and the Danish National Patient Registry (DNPR)[4] for intellectual disability (ID, ICD10: F70-F79; ICD8: 311-315, n=6,969) and epilepsy (ICD10: G40; ICD8: 345 (excl. 345.29), n=4,796).

Samples and genotyping

For all individuals within the iPSYCH case-cohort, DNA was extracted and whole-genome amplified from available neonatal blood spots retrieved from the Danish Neonatal Screening Biobank (DNSB),[5] and genotyped with Illumina genotyping arrays (Illumina, San Diego, CA, USA). The iPSYCH2015 case-cohort is an update and expansion of the study base of the iPSYCH2012 case-cohort, described in detail elsewhere[6]. The sampling and genotyping of additional samples (iPSYCH2015i, which when combined with iPSYCH2012 constitute the complete iPSYCH2015 case-cohort) differed in several ways as detailed elsewhere;[1] most importantly, iPSYCH2015i samples were genotyped using the Global Screening Array v2, whereas iPSYCH2012 samples had been genotyped using the PsychArray V1.0[6]. Single nucleotide polymorphism (SNP) genotype calling and quality control were performed using Illumina's GenTrain software tool for all samples that could be successfully identified and extracted from DNSB (95.5%). The extraction of B-allele frequency as well as intensity for each probe was performed using Illumina GenomeStudio. Samples with a genotyping call rate lower than 95% or unexplained genotype-estimated sex discordance with the Danish Civil Registration System[2] were excluded from further analysis.[1]

CNV calling and quality control analysis

The selection of rCNV loci into this study was largely based on the 54 CNVs studied in the UKB by Crawford *et al*.[7] with the following exceptions: NRXN1, NPHP1, CRYL1, and CHRNA7, as we considered these either too small to reliably detect in our dataset or to be CNVs of diverse/non-recurrent nature. Also, we did not test duplications spanning BP3-BP5 on 15q11-q13 specifically but instead applied a set of hierarchical rules to deal with rCNVs overlapping more than one adjacent recurrent CNV loci. We then cross-referenced the list of loci with the ClinGen database (https://clinicalgenome.org)[8] and removed those loci that were not included in the ClinGen list of recurrent CNVs or listed with "No Evidence" for neither haploinsufficiency nor triplosensitivity.[9] This yielded 27 distinct rCNV loci, in which we searched for deletions and duplications spanning at least ⅔ of the defined boundaries, as further outlined below and in eTable1.

© 2024 Vaez M et al. JAMA Psychiatry.

Intensity file preprocessing and CNV calling

Raw genotype data in the form of Illumina intensity files were stored on a secure partition on the HPC cluster GenomeDK (https://genome.au.dk/). We retrieved a total of 128,225 intensity files that could be used in the CNV calling pipeline. A small subset of samples had been genotyped more than once, and for these samples, we only kept PennCNV[10] calls from the intensity file with lower log-R-ratio standard deviation (LRR-SD; see below). In total, 123,377 unique iPSYCH2015 samples from the case-cohort design (Methods) were available for CNV analysis. To reduce probe intensity noise and erroneous CNV calling, we filtered the intensity files from each genotyping array type separately to include only biallelic autosomal SNPs mapping uniquely to the Haplotype Reference Consortium (HRC)[11] hg19 reference map, with a minor allele frequency of at least 0.1%, which yielded 280,700 and 509,754 probes for the PsychArray and GSA, respectively. Next, CNV calling was performed using PennCNV[10] in batches of 1,200 samples, using Population Frequency of B allele (PFB) files based on the actual B allele frequencies (BAF) in each batch. We used the default hidden Markov model (HMM) file provided by PennCNV[10] and produced array-specific GC-content (GCC) files based on the file "hg19.gc5Base.txt.gz" downloaded from the Genome browser of the University of California in Santa Cruz (https://genome.ucsc.edu/). Initial PennCNV calls were obtained with the script "*detect_cnv.pl*" setting minimum number of probes (--minsnp) at 5, and minimum length (--minlength) at 1000 bp. We then merged adjacent calls, with the PennCNV script "*clean_cnv.pl*" using the settings "*--fraction 0.2 --bp*" whereby two calls are merged if the gap between them corresponds to less than 20% of the combined length of the calls in terms of base pairs.

Subsetting and filtering CNVs at target recurrent CNV (rCNV) loci

PennCNV calls and sample QC files (containing LRR- and BAF-derived measures for each sample informing about overall sample quality) were imported into *R*, version 4.0.5. Subsetting and filtering of calls for the current study was then performed using the in-house developed *R* package *QCtreeCNV* (https://github.com/SinomeM/QctreeCNV). A general overview of the filtering steps and the *QCtreeCNV* pipeline is provided elsewhere[12]. First, we selected the calls in the 27 target autosomal loci (eTable1) and merged remaining adjacent calls in each sample using the function "*QCtreeCNV::select_stitch_calls()*" with default values (minsnp = 20, maxgap = 0.5, minoverlap = 0.2) for all loci except WBS and 22q11.2 where required minimum overlap was increased to 0.35. Also, the required minimum number of probes was reduced from 20 to 15 at four loci (TAR, 2q21.1, 16p12.1, and 16p11.2d) after we found upon initial visual inspection that a substantial fraction of true calls at these loci (ranging from 5% for 16p12.1 to nearly half of true 16p11.2d calls) involved 20-24 probes. We then used the function "*QCtreeCNV::qctree()*" to select putative carriers to visually inspect, using default values for all parameters except "*st5maxlogr1=0.55*".

Sample filtering and visual inspection

The distribution of per-sample quality measures; standard deviation of LRR (LRR-SD), BAF-drift, and GC-wave factor (GCWF), is shown in eFigure1. We discarded samples exceeding any of the three following threshold values; LRR-SD ≥ 0.35, BAF-drift ≥ 0.005 and/or |GCWF| ≥ 0.02. This resulted in the removal of 3,130 samples, corresponding to 2.5%. After removal of these samples and the filtering of rCNV calls as described above, we plotted LRR and BAF values for each of the 11,890 remaining rCNV calls. Each plot was then inspected by the senior author twice (to derive intra-rater reliability estimates) and by at least one of the two primary analysts, using *DeepEye*, an in-house developed graphical interface, designed to facilitate fast rating of CNV calls and accurate storage thereof, described in detail elsewhere[12]. Each call was rated as either true (T), false (F) or unknown (U). All calls with disagreement across the 3-4 ratings of the 2-3 raters were rated again jointly by all three raters to reach a consensus. We considered as true, those calls that had a LRR and BAF pattern consistent with a deletion or duplication (or, in some instances, triplication) overlapping at least 2/3 of the locus in question, irrespective of the actual boundaries of the putative PennCNV calls.

© 2024 Vaez M et al. JAMA Psychiatry.

CNV grouping and initial quality control

We split the 27 loci into four groups depending on locus size, while keeping the 15q11.2 locus as a separate fifth group, as rCNVs at this locus are by far the most prevalent, both among deletions and duplications (for the locus size ranges see eTable1). Some of the loci are adjacent to another locus or loci (eTable1) and many true rCNV calls were found to span more than one locus. To account for this, we applied hierarchical filtering of true calls by removing (only in instances involving same rCNV type): (a) true 15q11.2 and 15q13.3 calls in samples with a true Prader-Willi/Angelman Syndrome (PWAS) call; (b) true 22q11.2b and 22q11.2d calls in samples with a true 22q11.2 call; (c) true 16p12.1 and 16p11.2d calls in samples with a true 16p11.2 call; (d) true TAR calls in samples with a true 1q21.1 call; and (e) true 7q11.23d calls in samples with a true WBS call (in total 233 redundant calls were removed).

To adhere to legislation regarding protection of personal-level data in research of nation-wide registers and biobanks, which limits detailing of results for ultra-rare exposures or outcomes, we excluded any locus where the population-based prevalence of deletions and duplications combined (estimated through the same approach as outlined below, in the first subsection of the "Statistical analysis" section) corresponded to less than 0.01%. This resulted in the removal of nine out of 27 loci from further analysis (eTable1) and reduced the number of true rCNV calls to 3446, corresponding to 97.2% of the total number of true calls across all 27 loci .

We then applied rigorous quality control to verify the accuracy and validity of the rCNV calling and subsequent visual inspection at the remaining 18 loci. First, we derived the fraction of true calls at each locus and estimated the intra- and inter-rater reliability. Out of 6,569 calls, 3,446 were found to be true, corresponding to 52.5%, a fraction which varied substantially across rCNVs, from 6.1% for 2q11.2-dup to 100% for several deletions and duplications (eTable2). In some cases, low true call rates are explained by a high number of spurious small calls that would have been avoided by setting the requirement of minimum overlap with target loci higher than the 20%-35% threshold that we applied. The reason we set this criterion so loose is that some true calls are only partially captured by PennCNV, and some of these would have been missed by a stricter criterion; this is especially true for duplications. However, low true call rates can also be indicative of higher-than-average noise level in LRR and BAF at some loci, which could affect both the PennCNV performance in detecting true calls, and our performance in the subsequent visual inspection. To explore this possibility, we tested the correlation between true call rates and the magnitude of difference in estimated prevalence of rCNVs between our study and the UKB. As shown in eFigure2, there was no indication of such correlation (Pearson's correlation test; R = -0.11, P = 0.54), which corroborates results of other analyses into possible effects of sample noise (LRR-SD), genotyping array and genotyping wave on rCNV prevalence (see next subsection).

Next, we studied whether the fraction of U calls or inter-rater reliability (IRR) differed by sample LRR-SD, rCNV type, locus group, and genotyping array (eTable3). Unsurprisingly, the fraction of calls rated as unknown (U) increased with increased LRR-SD, while the IRR decreased with increased LRR-SD, with both trends most noticeably observed for the small (S) and medium-sized (M) loci. This indicates reduced confidence in our rating of calls in (a) samples with higher compared to lower LRR-SD, and (b) smaller compared to larger loci. Also, the fraction of unknown calls and the IRR differed significantly between genotyping arrays (see eTable3).

Population-based rCNV prevalence by LRR-SD, and genotyping array and wave

To study whether this association between (a) LRR-SD, locus size and genotyping array, and (b) confidence in initial rating of calls had affected the validity of our final consensus ratings, we tested for association between these variables and the population-based prevalence of true calls (i.e., calls that we consider true based on our visual inspection). The analyses were done in *R* using the *survey* package[13] functions *svydesign()*, *svyciprop()* and *svyglm()*, and applying finite population correction (fpc) to account for oversampling of cases in the case-cohort sample, as explained in the first subsection of the next methods section (statistical analysis). The *svyglm()* was used to assess the potential effect of sample LRR-SD and genotyping array (PA or GSA) on the prevalence of deletions and duplications separately. We found no evidence for an effect of either LRR-SD or genotyping array on deletion or duplication prevalence, neither overall nor when grouping loci based on size nor at any specific locus, except for 2q11.2 deletions where prevalence was higher in samples genotyped on the PsychArray (0.009% vs 0.001%

© 2024 Vaez M et al. JAMA Psychiatry.

on GSA, P_{FDR}=0.011, eTable4). However, at such low ranges, the model is vulnerable to small changes in carrier counts, and since the overall prevalence is roughly the same as in the UKB (0.006% vs 0.007%), we decided not to exclude this rCNV from further analysis.

Finally, as the iPSYCH2012 samples were genotyped in different genotyping waves, we investigated how the proportion of cases and samples dropped through QC as well as overall deletion and duplication prevalence were distributed across genotyping waves (eTable5). In short, all genotyping waves included both affected and unaffected subjects and samples lost to QC. A qucik comparison of numerical values across the table identified one outlier wave for each, proportion of cases (w25 with 4% of cases vs 67% average) and QC-dropouts (w23 with 8% dropout vs 2% average), but neither was found to significantly differ from remaining iPSYCH2012 waves in either deletion or duplication prevalence (pDel = 1.12% and 1.29% vs 1.01%; P = 0.69 and 0.49; and pDup = 1.10% and 1.18% vs 1.01%; P = 0.40 and 0.70; for w23 and w25 vs other iPSYCH2012 waves, respectively). Additionally, we provide the breakdown of the number of samples for all tested outcomes by genotyping array (eTable6).

Statistical analysis

Estimating and comparing rCNV prevalence:

We calculated population-based rCNV prevalence (with CI95%) from the full iPSYCH2015 case-cohort using the *svydesign()* and *svyciprop()* functions from the *survey*[13] package in R, with finite population correction (fpc) to account for oversampling of cases. Briefly, we divided the post-QC number of (a) case individuals (79,535) and (b) individuals from the randomly drawn population subcohort (43,311) with the total number of corresponding individuals in the source population (92,531 and 1,657,449) to derive the sampled population fractions; 0.85955 and 0.02613, respectively. Samples from overlapping individuals (cases-in-subcohort) were assigned the case population fraction (0.85955). The survey model then uses these weights to derive estimates representative of the entire source population (with an effective population-based test sample size corresponding to 45,609 individuals). We compared the overall and per-locus prevalence of deletions and duplications with a Welch's test of difference between two measures assuming unequal variance. Briefly, we defined the difference; $d = abs(log(p_{DEL}/p_{DUP}))$, the standard error of the difference; $SEd = \sqrt{(SE_{DEL}^2+SE_{DUP}^2)}$, and the p-value; $P = 2*(1-pnorm(d/SEd))$, where p_{DEL} and SE_{DEL}, and p_{DUP} and SE_{DUP}, indicate the prevalence and standard error of prevalence retrieved with *svyciprop()* for deletions and duplications, respectively. We calculated rCNV prevalence in the UKB directly from carrier counts provided in Crawford *et al.*[6] and CI95% calculated as follows: *CI95% = qbeta(c(0.05/2,1-0.05/2), nCarrier+0.5, nTotal-nCarrier+0.5)*, where *nCarrier* and *nTotal* indicate the number of carriers of the rCNV and the total number of assessed samples (421,268), respectively. We then compared the rCNV prevalence in iPSYCH2015 and UKB with a Welch's test, as described above. P-values were in both instances adjusted for multiple comparisons with a false discovery rate (FDR) method, using the *p.adjust* function of the (default) *stats* package in R.

Estimating rCNV-associated risk of iPSYCH disorders with survival analysis:

We used weighted Cox proportional hazard (CPH) models from the *survival*[14] package in R to estimate the rCNV-associated risk of the five psychiatric disorders targeted by the iPSYCH2015 case-cohort design (ADHD, ASD, BPD, MDD, and SSD), as well as of the broader diagnosis groups; any affective disorder and any iPSYCH disorder, and the narrower (compared to SSD) diagnosis of schizophrenia (ICD10; F20). The outcome in the CPH models was the age at first hospital diagnosis with the index disorder, age at censoring or age at death - whichever came first - and the exposure was carrier status (i.e., having deletion or duplication, respectively, versus normal copy number) for the rCNV. All models were sex-stratified. Subjects were censored if they had not received an index diagnosis by the end of the follow-up period (December 31st, 2015), emigrated, or otherwise had been lost to follow-up. To obtain unbiased population-based estimates, the inverse probability of sampling (IPS) weights were used as introduced by Barlow *et al.*[15] The SEs of regression coefficients were computed by a robust estimator to derive CI95% and test for the significance of rCNV-associated HRs.

© 2024 Vaez M et al. JAMA Psychiatry.

Estimating omnibus rCNV-associated risk of the four main iPSYCH2015 disorders:

We used generalised linear models (GLMs) to assess the overall effect of rCNVs on the prediction of each of the four main iPSYCH2015 disorders (ADHD, ASD, MDD, and SSD) separately. For this analysis we defined a categorical variable with separate levels for each rCNV and "no rCNV" as reference (individuals carrying more than one rCNV were assigned the level corresponding to the rCNV with a higher risk estimate for "any iPSYCH2015 disorder"). Then for each disorder, a full model including this categorical rCNV carrier status as an independent variable in addition to AEF and biological sex (as determined at birth), was compared with a nested model including only AEF and biological sex. Both models included only individuals either diagnosed with the respective diagnosis or belonging to the random population subcohort. A likelihood ratio test (LRT) was used to test the omnibus rCNV effect by comparing the full model with the nested one.

Comparison of rCNV-associated risk across diagnoses:

We implemented generalised estimating equation (GEE) models using *glmgee()* function from *glmtoolbox*[16] in R to test for overall and/or rCNV-specific differences in associated risk between pairs of diagnoses (ASD, ADHD, and SSD). We limited each analysis to those individuals who were part of the random population subcohort or diagnosed with either of the two compared diagnoses and allocated two lines to each sample ID in the data structure to account for the possibility of having been diagnosed with both disorders. Thus, the binary case outcome in the model was independent between diagnoses for each included study subject. For each pairwise comparison, two GEE models (full and nested) were constructed, with case status as outcome and clustering on sample ID. Independent categorical variables were rCNV and diagnosis (i.e. indicating which of the two compared disorders each line's outcome value belonged to for each study subject, in each comparison the diagnosis with a larger case sample size was assigned as reference level), along with sex and AEF. In the analysis of overall rCNV-associated differences between diagnoses, rCNV status was defined as binary carrier status for "any rCNV", while in the analysis of rCNV-specific cross-diagnosis differences, a categorical variable with separate levels for each rCNV was specified for rCNV status with "no rCNV" as the reference (if individuals carried more than one rCNV, we assigned them the level of the rCNV with higher associated estimate for "any iPSYCH disorder"). In addition to the variables specified above, the full models included an interaction term between rCNV status (binary or categorical) and diagnosis, whereas the nested models did not. We then used anova test from the *glmtoolbox* package (with *test="score"* and *varest="model"*) to compare each full model with its respective nested model. Furthermore, to extract significant coefficient of interaction between rCNVs and the diagnosis, we performed *post hoc* analysis on summary results of the GEE models (i.e the model that included the interaction term) by applying FDR correction on the p-values corresponding to all coefficients in the model outputs.

Analysis of locus type effect on rCNV-associated risk across the diagnoses:

To investigate the rCNV dosage type effect (i.e., deletion v. duplication) on rCNV pathogenicity across ASD, ADHD, and SSD, we again applied GEE models similarly as described above, although only among rCNV carriers. To account for multi-diagnoses individuals, three rows were assigned to each individual id in the data structure corresponding to the three included diagnoses, and the binary case status was defined in each row independently based on whether the rCNV carrier had any of the diagnoses. The GEE models included diagnosis, rCNV type (del v. dup), and locus of the corresponding rCNV carrier as independent categorical variables, to predict the case status as the model outcome (binary variable), while adjusting for AEF and sex and clustering on ids. The reference categories for rCNV type, locus, and diagnosis as categorical variables were set as deletion, 15q11.2, and ADHD respectively in each model. To evaluate overall locus type effect across three diagnoses, we compared the full model with a nested model excluding rCNV type from the covariates by performing ANOVA test from the same package and default mentioned above. Additionally, to test locus-specific rCNV type effects, we built another model containing an interaction between rCNV type and locus and compared it with the base model using ANOVA test.

Analysis of locus features and rCNV prevalence as predictors of effect size:

© 2024 Vaez M et al. JAMA Psychiatry.

For analysis of locus features' effect on rCNV pathogenicity, we constructed two GEE models among all rCNV carriers. The first model contained loss-of-function observed/expected upper fraction (LOEUF) score and locus size (see eTable1), while the second model contained both rCNV iPSYCH prevalence and iPSYCH/UKB prevalence ratio (see eTable7) as independent variables to predict the case status (binary outcome) separately. Both models included AEF and sex as covariates and sample ID as clustering variable. In both models, the test variables (LOEUF score and size, iPSYCH prevalence, and iPSYCH/UKB prevalence ratio, respectively) were log-transformed. LOEUF scores were obtained from the GnomAD database (v2.1.1)[17] for all genes overlapped at least 50% by an rCNV loci in our study (see full list in eTable12). The LOEUF scores in GnomAD are constructed such that lower scores indicate more gene constraint (i.e. intolerance to loss-of-function mutations); therefore, we first inverted the scores, and then derived the sum of the inverted scores (1/LOEUF) for all genes at each rCNV locus.

Estimating rCNV-associated risk of ID and epilepsy with survival analysis

To study ID and epilepsy, we fitted weighted CPH models with age at the first hospital diagnosis (otherwise censoring date) as the primary outcome on the entire case-cohort sample. Considering the frequent comorbidity between ID and epilepsy with psychiatric outcomes, we assigned IPS weights to the individuals depending on whether they belonged to the case or cohort component of iPSYCH2015 in a similar way as for the primary outcomes.[15] All the analyses included sex as the stratification variable and rCNV status as the independent variable, which was coded as described in primary analyses of iPSYCH outcomes.

Sensitivity analyses using generalised linear models for all the studied outcomes:

Sensitivity analysis was performed utilising generalised linear models (GLMs) in parallel to CPH models to compute rCNV-associated ORs for all the studied outcomes. When studying psychiatric diagnoses, only the individuals who had the corresponding outcome or were in the random cohort were included in the analyses, with the diagnosis as the binary outcome and rCNV as independent variable in each model accounting for AEF and sex of the individuals and genotyping array (i.e whether individual was genotyped on PsychArray or GSA).

All statistical analyses were conducted in R[18], version 4.2.3.

© 2024 Vaez M et al. JAMA Psychiatry.

eFigure1: Per-sample CNV-calling quality measures by genotyping array

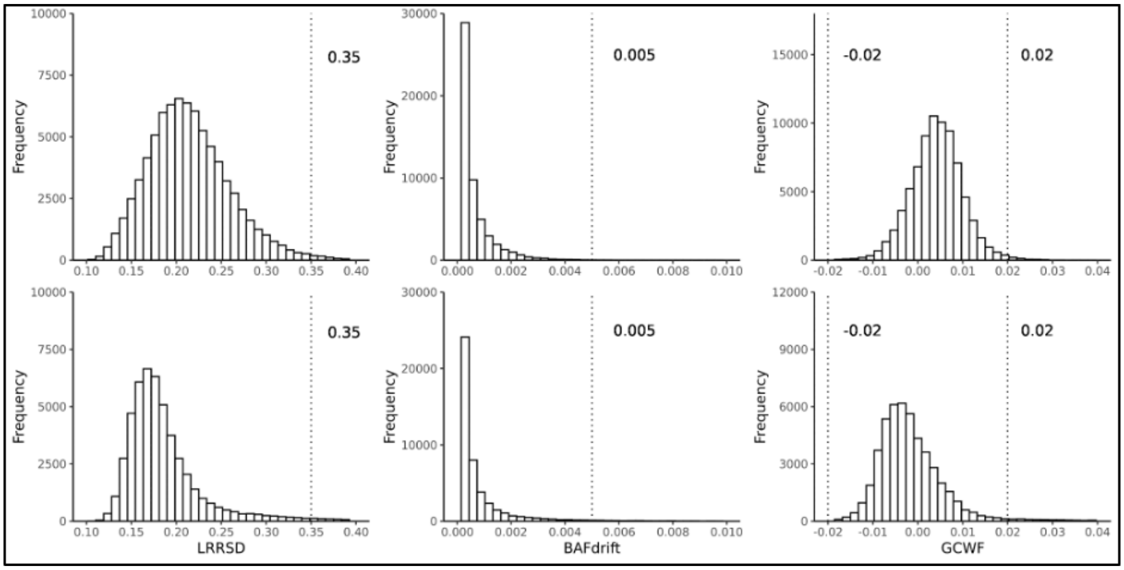

Distribution of per-sample LRR-SD (left), BAF-drift (middle) and GCWF (right) in iPSYCH2015 (Upper row: Samples genotyped with Illumina PsychArray in the initial iPSYCH2012 study[5]. Lower row: Samples genotyped with Illumina GSA in the iPSYCH2015i expansion, which together with iPSYCH2012 samples constitutes iPSYCH2015[1]). Vertical lines and adjacent numeric labels indicate the cutoff values for each measure used to discard outlying samples with intensity data of too low quality to reliably call rCNVs. Samples falling outside of any cutoff value were excluded.

eFigure2: rCNV prevalence difference with UKB vs initial PennCNV specificity

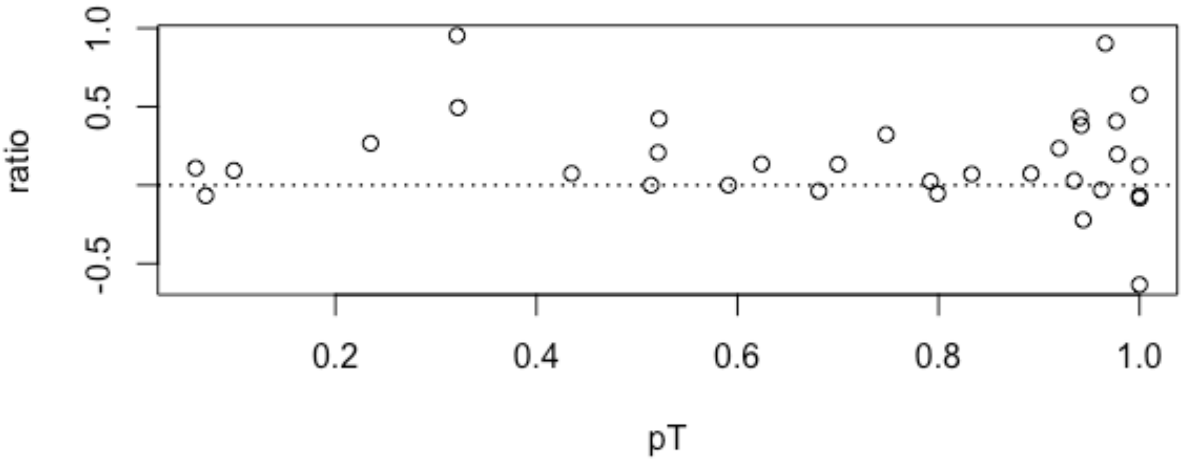

We inspected all PennCNV[10] calls that included at least 20 probes and overlapped at least 20% of any target locus, and determined which calls were "true CNV, covering at least 2/3 of the target region". Then, 12 out 27 rCNV loci were excluded due to very low (<0.01%) population-based prevalence of CNVs. As a low proportion of true calls can indicate low data quality that could affect PennCNV's and our ability to detect and determine true CNVs, we tested whether the proportion of true calls (pT, x-axis) at each of the 36 remaining CNVs (deletions and duplications at 18 rCNV loci) correlates with the prevalence ratio between our study and that of the UK biobank (ratio, y-axis); the ratio is log-transformed so "0" means the prevalence is the same in both studies. We did not find that the two measures were correlated (Pearson's correlation test; R = -0.11, P = 0.54).

© 2024 Vaez M et al. JAMA Psychiatry.

eFigure3: rCNVs and risk of psychiatric disorders in iPSYCH2015 (by locus)

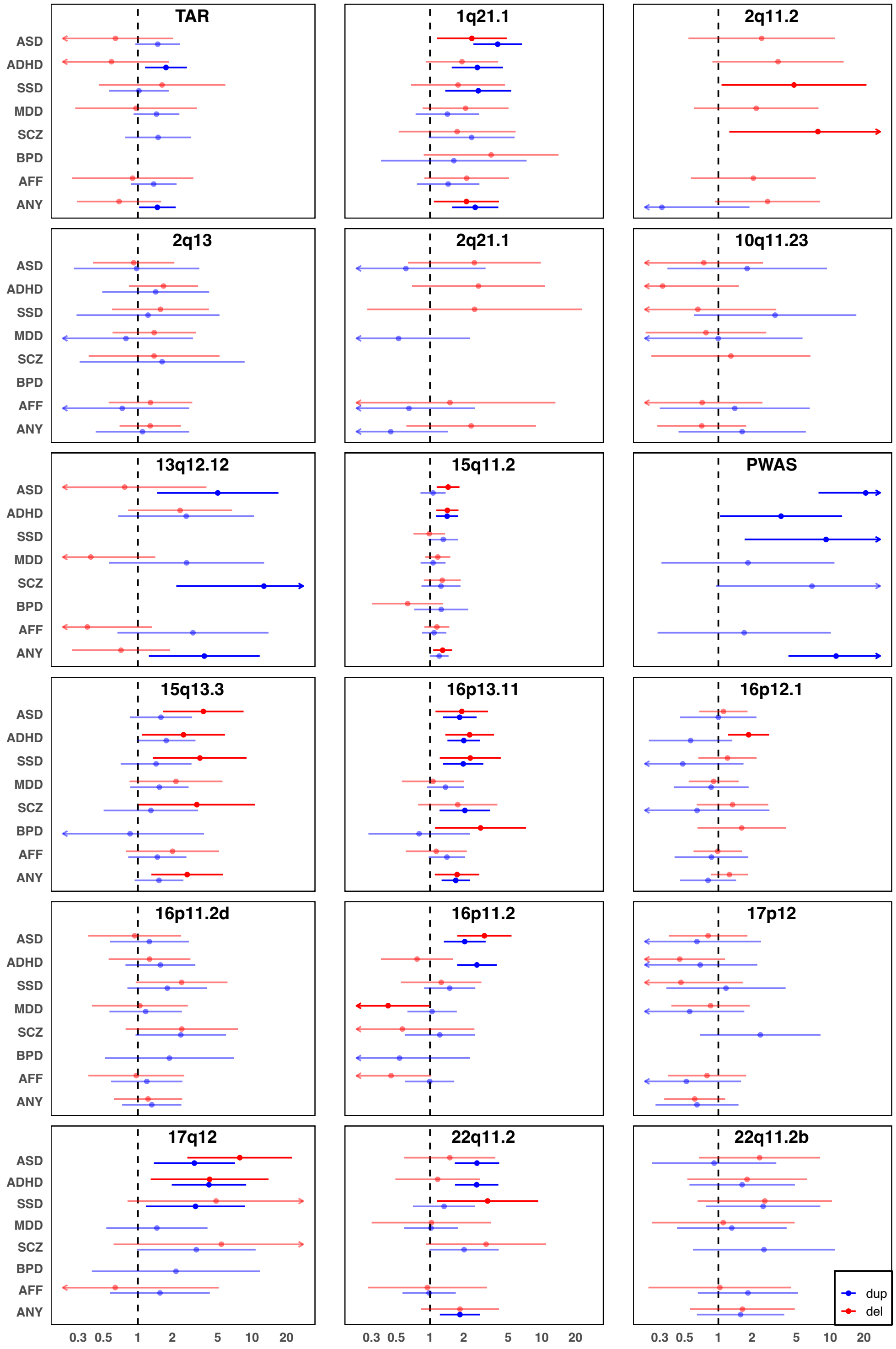

HRs and CI95% for deletions and duplications are indicated in red and blue, respectively, and bolded when CI95% do not overlap HR=1. ADHD; attention-deficit hyperactivity disorder, ASD; autism spectrum disorder, MDD; major depressive disorder, SSD; schizophrenia spectrum disorder, SCZ; schizophrenia (ICD10: F20), BPD; bipolar disorder, AFF; any affective disorder, ANY; any iPSYCH disorder. Any iPSYCH disorder is defined as being diagnosed with any of index disorders in iPSYCH2015 case-cohort. Comparisons involving <2 case carriers, or where the CPH model failed a test of proportionality of hazards, were excluded (indicated by empty lines).

© 2024 Vaez M et al. JAMA Psychiatry.

eFigure4: Comparison of risk estimates between iPSYCH2015 and case-control studies

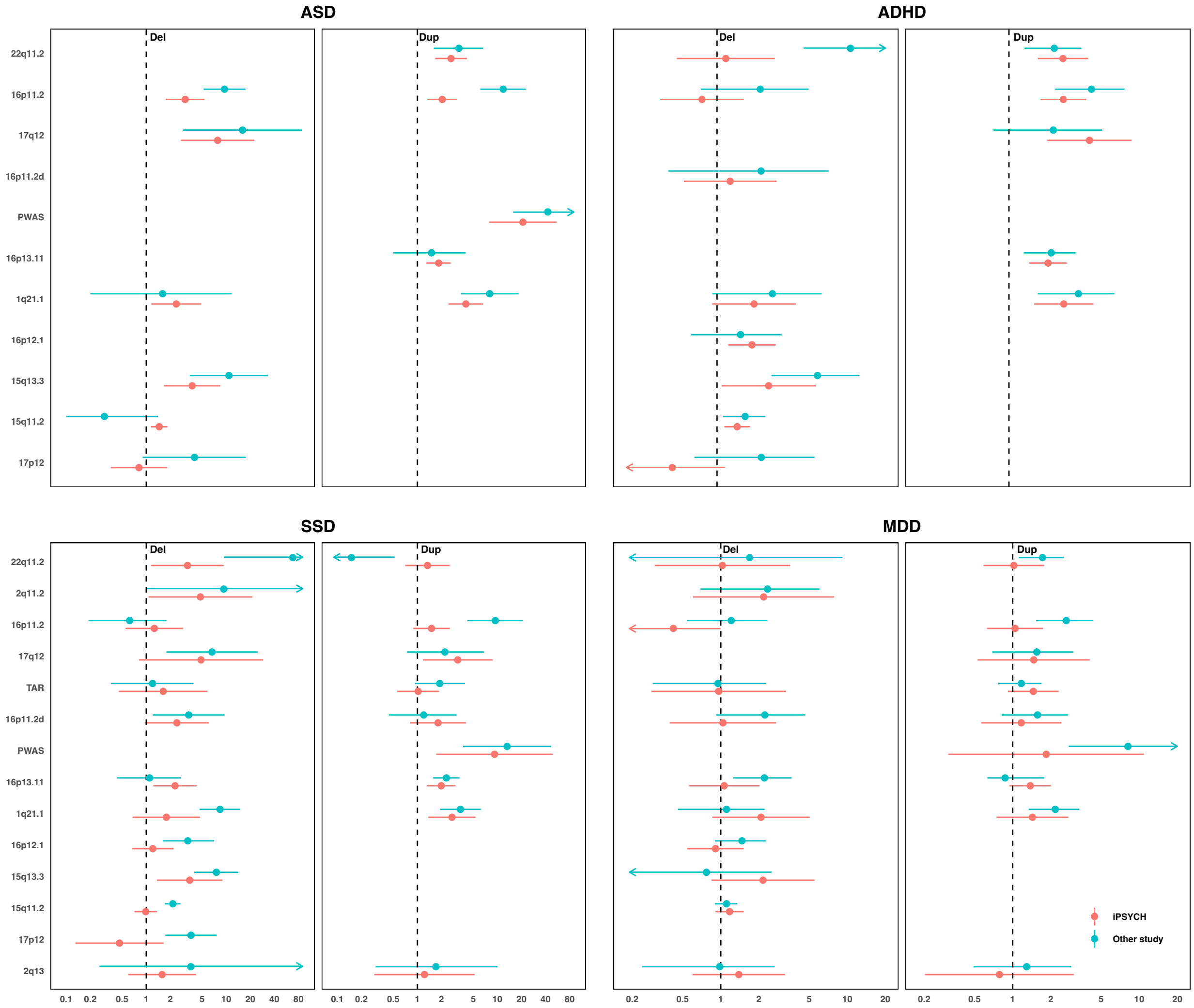

We compared the population-valid risk of ASD, ADHD, SSD, and MDD estimated for iPSYCH2015 (red) with previously published risk estimates (blue) associated with rCNVs across the 18 loci assessed in this study. The number of rCNVs with available published risk estimates ranged from 11 for ASD[19] to 13 for ADHD[20], 20 for MDD[21], and 22 for SSD[22-24] (eTable9). Risk estimates for iPSYCH2015 are hazard ratios (HR), while those for the compared case-control studies are odds ratios (OR). Error bars indicate 95% confidence intervals. The x-axis is log-scaled and differs in range between the two columns. Note that the loci shown on y-axis vary between the first row (ASD, ADHD) and the second row (SSD, MDD). Only HRs with comparable ORs from other studies are displayed on the plots. Note also that the diagnostic criteria for specific disorders may differ between iPSYCH2015 and the respective comparison case-control study. ASD: autism spectrum disorder, ADHD: attention-deficit/hyperactivity disorder, SSD: schizophrenia spectrum disorder, MDD: major depressive disorder.

© 2024 Vaez M et al. JAMA Psychiatry.

eFigure5: Coefficients of contrasts in rCNV-associated risk between iPSYCH disorders

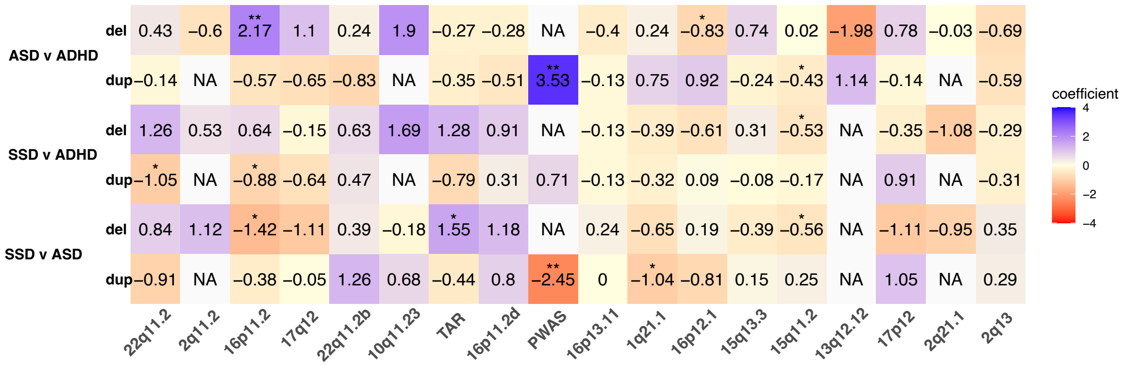

We constructed a heatmap of the coefficients from the rCNV-by-diagnosis interaction derived from GEE models for each pairwise comparison across all rCNVs. Significant coefficients are indicated with asterisks (P_{FDR}<0.05 (*), P_{FDR}<0.01 (**)). rCNV loci are ordered on the x-axis from left to right by decreasing sum LOEUF score,[17] with coefficients for deletions and duplications shown in separate rows for each pairwise diagnosis comparison. Only rCNVs with available HRs for both compared diagnoses were included in each GEE analysis.

eFigure6: rCNV-associated risk by prevalence and by iPSYCH/UKB prevalence ratio

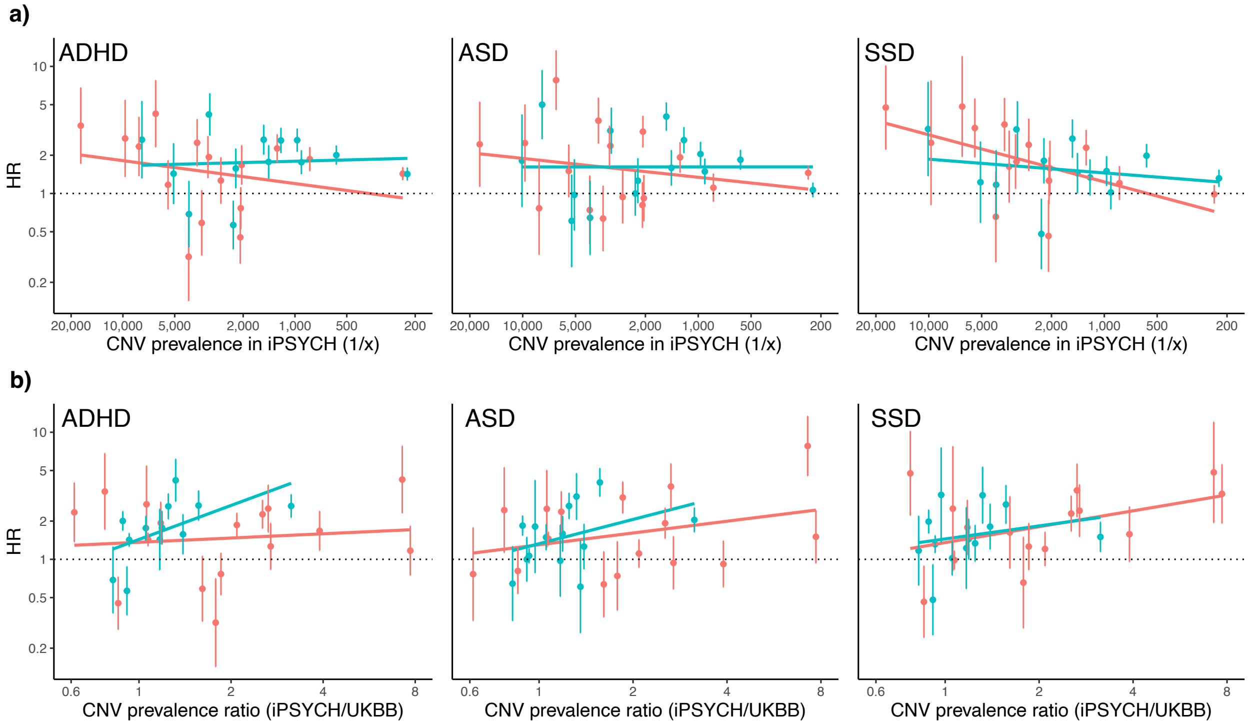

Hazard ratios (HR) with error bars indicating standard errors (SE) for deletions (light red) and duplications (iris blue) associated with ADHD (left), ASD (middle), and SSD (right) are plotted against **a)** population prevalence in iPSYCH2015 and **b)** the population prevalence ratio between iPSYCH2015 and UKB[7]. PWAS-dup was excluded because of outlying effect size, and 13q12.12-dup was excluded from **d)** owing to the outlying low iPSYCH2015/UKB prevalence ratio. The plots include fitted trend lines (created with geom_smooth). The overall trend across all three psychiatric outcomes for the two CNV prevalence-related features was assessed with generalised estimating equation (GEE) models including all rCNV carriers (eMethods), and significant positive association was found for the prevalence ratio between iPSYCH2015 and UKB (P=0.0086), but no association was found for CNV prevalence in iPSYCH2015 (P=0.31).

© 2024 Vaez M et al. JAMA Psychiatry.

eFigure7: rCNV-associated risk of other brain disorders in iPSYCH2015

ID

Locus	del: Hazard Ratio (95% CI)	dup: Hazard Ratio (95% CI)
22q11.2	18.46 (7.98–42.70)	3.80 (2.15–6.71)
2q11.2	13.43 (2.97–60.73)	NA
16p11.2	5.14 (2.67–9.91)	4.43 (2.76–7.09)
17q12	16.07 (5.90–43.78)	2.96 (1.06–8.25)
22q11.2b	1.66 (0.20–13.83)	NA
10q11.23	0.67 (0.08–5.29)	1.97 (0.22–17.72)
TAR	1.18 (0.26–5.29)	1.18 (0.55–2.51)
16p11.2d	2.76 (1.07–7.11)	1.86 (0.69–5.01)
PWAS	NA	46.45 (20.05–107.64)
16p13.11	6.12 (3.63–10.33)	2.04 (1.24–3.35)
1q21.1	5.38 (2.40–12.09)	3.14 (1.59–6.20)
16p12.1	1.49 (0.75–2.96)	1.24 (0.36–4.26)
15q13.3	10.40 (5.03–21.51)	2.28 (1.04–5.00)
15q11.2	1.46 (1.02–2.07)	1.25 (0.86–1.83)
13q12.12	2.40 (0.48–12.13)	2.09 (0.25–17.29)
17p12	0.33 (0.04–2.49)	2.80 (0.88–8.86)
2q21.1	4.67 (1.27–17.21)	2.05 (0.41–10.08)
2q13	1.54 (0.52–4.56)	0.83 (0.10–6.68)

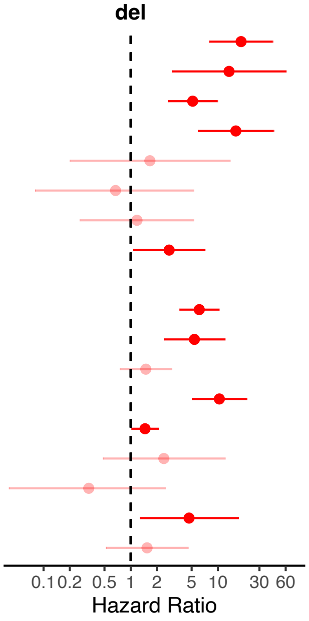

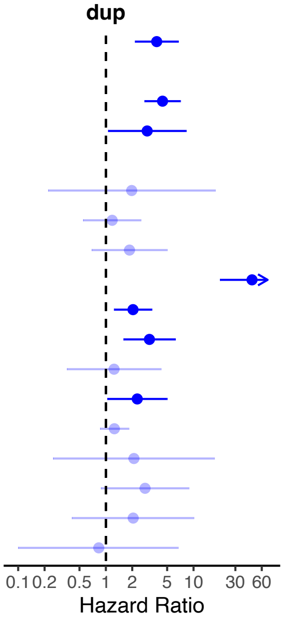

Epilepsy

Locus	del: Hazard Ratio (95% CI)	dup: Hazard Ratio (95% CI)
22q11.2	11.43 (5.36–24.35)	1.97 (0.85–4.60)
2q11.2	9.52 (2.42–37.40)	NA
16p11.2	5.47 (2.59–11.57)	2.35 (1.20–4.62)
17q12	NA	2.01 (0.48–8.45)
22q11.2b	6.38 (2.03–20.09)	1.48 (0.20–11.15)
10q11.23	NA	3.15 (0.36–27.78)
TAR	NA	1.49 (0.65–3.42)
16p11.2d	5.26 (2.17–12.75)	1.20 (0.28–5.07)
PWAS	NA	15.11 (7.38–30.91)
16p13.11	10.03 (6.60–15.24)	0.50 (0.16–1.56)
1q21.1	1.63 (0.41–6.47)	1.79 (0.66–4.88)
16p12.1	1.64 (0.76–3.56)	0.63 (0.09–4.65)
15q13.3	7.33 (3.32–16.16)	1.70 (0.62–4.62)
15q11.2	1.90 (1.29–2.79)	0.91 (0.53–1.55)
13q12.12	3.20 (0.65–15.77)	3.24 (0.59–17.67)
17p12	NA	3.35 (1.00–11.22)
2q21.1	4.17 (0.48–36.10)	NA
2q13	0.64 (0.09–4.69)	NA

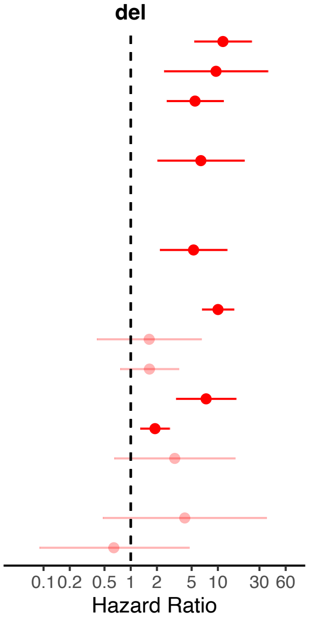

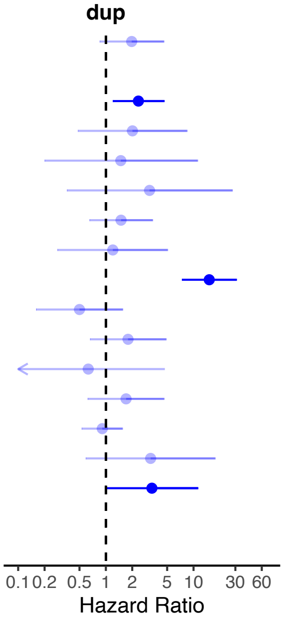

rCNV-associated hazard ratios (HR) and 95% confidence intervals (CI95%) were derived for intellectual disability and epilepsy from available hospital diagnoses using a sex-stratified Cox proportional hazards (CPH) model with inverse probability of sampling (IPS) weights. Comparisons involving <2 case carriers, or where the CPH model failed a test of proportionality of hazards, were excluded (indicated by "NA"). We used IPS weights corresponding to overall case status for any of the iPSYCH2015 index psychiatric disorders, and therefore the HRs are not population-unbiassed for these two disorders (intellectual disability and epilepsy) as they are for the index psychiatric disorders. ID; intellectual disability.

© 2024 Vaez M et al. JAMA Psychiatry.

eTable1: rCNV loci selected for study in the iPSYCH2015 case-cohort sample

rCNV locus	Breakpoints/gene[a]	Hg19 range (Chr:Mb)	Size (Mb)	Locus group[a]	n Probes (PA/GSA)[b]	Genes (n)[c]	LOEUF (sum^{-1})[d]	ClinGen HI/TS[e]	ClinGen Disease[f]	Filtered in QC[g]
TAR	BP2 - BP3	1:145.39-145.81	0.42	S	55/56	16	22.2	1/1	loss	
1q21.1	BP3 - BP4	1:146.53-147.39	0.86	M	135/177	7	9.1	3/3	loss/gain	
2q11.2	ARID5A/TMEM127	2:96.74-97.68	0.94	M	82/108	18	43.8	3/1		
2q13	BCL2L11	2:111.39-112.01	0.62	M	71/131	3	5.0	2/2		
2q21.1	ARHGEF4, GPR148	2:131.48-131.93	0.45	S	31/68	4	5.1	1/0		
3q29	DLG1	3:195.72-197.35	1.63	L	286/341	21	41.3	3/1	loss/gain	yes
WBS	ELN	7:72.74-74.14	1.40	L	118/189	23	50.2	3/3	loss/gain	yes
7q11.23d	HIP1, YWHAG	7:75.14-76.06	0.92	M	93/168	12	17.0	2/1		yes
8p23.1	GATA4	8:8.10-11.87	3.77	XL	673/1,026	27	27.5	3/2		yes
10q11.23	LCR C - LCR D	10:49.39-51.06	1.67	L	287/345	17	23.5	1/1		
10q23	BMPR1A	10:82.05-88.93	6.88	XL	660/1,165	24	39.9	3/1		yes
13q12.12	SACS, SGCG	13:23.56-24.88	1.32	L	273/403	6	8.0	0/40		
15q11.2	BP1 - BP2	15:22.81-23.09	0.28	15q11.2	49/66	4	8.4	3/40		
PWAS	BP2 - BP3	15:24.82-28.39	3.57	XL	471/871	8	19.0	3/3	loss/gain	
15q13.3	BP4 - BP5	15:31.08-32.46	1.38	L	153/253	6	8.7	3/1		
15q24	LCR A - LCR D	15:72.90-78.15	5.25	XL	460/701	64	112.3	3/1		yes
16p13.11	BP2 - BP3	16:15.51-16.29	0.78	M	125/213	8	19.0	3/2		
16p12.1	EEF2K, CDR2	16:21.95-22.43	0.48	S	47/50	8	9.0	2/0		
16p11.2d	BP2 - BP3	16:28.82-29.05	0.23	S	22/35	9	21.4	3/1	loss	
16p11.2	BP4 - BP5	16:29.65-30.20	0.55	S	50/53	28	42.9	3/3	loss/gain	
17p12	PMP22	17:14.14-15.43	1.29	L	237/406	6	6.5	3/3	loss/gain	
PLS	RAI1	17:16.81-20.21	3.40	XL	293/444	49	76.6	3/3	loss/gain	yes
17q11.2	NF1	17:29.12-30.27	1.15	M	101/154	14	26.3	3/2	loss/gain	yes
17q12	HNF1B	17:34.81-36.22	1.41	L	161/282	15	37.1	3/3	loss/gain	
22q11.2	proximal (A - B)	22:18.90-20.30	1.4	L	172/328	27	50.4	3/3	loss/gain	
22q11.2b	central (B/C - D)	22:20,71-21,47	0.76	M	86/141	15	24.2	2/0		
22q11.2d	distal type I (D - E/F)	22:21,92-23,65	1.73	L	166/287	20	30.6	3/0	loss	yes

[a]To test the effect of locus size in the QC, we grouped loci according to size (S; <0.6 Mb, M; 0.6-1.2 Mb, L; 1.2-1.8 Mb, XL; >3 Mb), keeping 15q11.2 separate given its high prevalence. [b]Number of probes used for rCNV calling from the Illumina PsychArray (PA) and Global Screening Array (GSA). [c]Number of genes overlapped >50% by the locus (full list in eTable 12). [d]Sum of LOEUF scores of all locus genes (per-gene scores were inverted as lower LOEUF indicates increased gene constraint, see details in eTable 12). [e]Assessed haploinsufficiency (HI) and triplosensitivity (TS) scores[9] in the ClinGen[8] database (clinicalgenome.org, accessed on 2024-02-20); 0=No evidence, 1=Little evidence, 2=Emerging evidence, 3=Sufficient evidence, 30=Autosomal recessive, 40=Dosage sensitivity unlikely. [f]Curated loss and/or gain disease registered in the ClinGen[8] database. [g]Loci with an estimated population-based prevalence of less than 1 in 10,000 individuals for deletions and duplications combined were excluded from further analysis.

© 2024 Vaez M et al. JAMA Psychiatry.

eTable2: Results of visual inspection of PennCNV calls across included rCNV loci

rCNV locus	Deletions					Duplications				
	Inspected Calls (n)[a]	True Calls (n)[b]	True Calls (%)[b]	Intra-RR (TvF|U)[c]	Inter-RR (TvF|U)[c]	Inspected Calls (n)[a]	True Calls (n)[b]	True Calls (%)[b]	Intra-RR (TvF|U)[c]	Inter-RR (TvF|U)[c]
TAR	48	25	52.1%	89.3%	93.8%	221	175	79.2%	98.1%	95.0%
1q21.1	74	66	89.2%	100%	100%	139	136	97.8%	100%	98.6%
2q11.2	241	17	7.1%	97.9%	79.7%	98	6	6.1%	100%	99.0%
2q13	57	57	100%	100%	96.5%	30	25	83.3%	100%	96.7%
2q21.1	35	18	51.4%	88.9%	82.9%	20	14	70.0%	100%	90.0%
10q11.23	25	23	92.0%	100%	96.0%	22	13	59.1%	100%	100%
13q12.12	18	17	94.4%	100%	94.4%	23	23	100%	100%	100%
15q11.2	651	609	93.5%	98.8%	98.3%	602	579	96.2%	99.0%	97.8%
PWAS	5	5	100%	100%	100%	52	49	94.2%	100%	98.1%
15q13.3	68	64	94.1%	100%	98.5%	239	104	43.5%	100%	99.6%
16p13.11	132	129	97.7%	99.2%	100%	368	294	79.9%	100%	96.7%
16p12.1	218	163	74.8%	95.2%	98.2%	69	47	68.1%	91.8%	81.2%
16p11.2d	92	48	52.2%	90.4%	85.9%	109	68	62.4%	92.3%	88.1%
16p11.2	345	81	23.5%	98.3%	86.4%	575	185	32.2%	98.3%	96.0%
17p12	45	45	100.0%	100%	100%	24	24	100%	100%	95.8%
17q12	29	28	96.6%	100%	100%	65	65	100%	100%	96.9%
22q11.2	112	36	32.1%	97.3%	97.3%	1,577	156	9.9%	99.8%	99.7%
22q11.2b	32	23	71.9%	100%	100%	109	29	26.6%	96.3%	96.3%

[a]After processing PennCNV calls through our QCtreeCNV pipeline, all putative calls overlapping at least 20% of a given rCNV region were verified by at least two analysts through visual inspection of intensity data. [b]We only considered calls true if log-R-ratio and B-allele frequency patterns were consistent with a deletion or duplication spanning at least 2/3 of a rCNV region. [c]Calls were assigned as; true (T), false (F) or unknown (U), and we derived intra- and inter-rater reliability rates for (T) v. (F or U) assignments.

eTable3: Factors affecting initial evaluation of rCNV calls

Predictor	Level	N (calls)	pU[a]	iIRR[a]	Anova model[b]
LRR-SD	0.10-0.15	327	0.003	0.015	pU; deviance = 210.2, P = 1.2×10^{-47}<br>iIRR; deviance = 207.5, P = 4.9×10^{-47}
	0.15-0.20	3,111	0.010	0.013	
	0.20-0.25	2,046	0.026	0.035	
	0.25-0.30	860	0.106	0.108	
	0.30-0.35	225	0.142	0.160	
Locus group	15q11.2	1,253	0.015	0.019	pU; deviance = 160.1, P = 2.5×10^{-25}<br>iIRR; deviance = 155.4, P = 2.1×10^{-24}
	S (<0.6 Mb)	1,732	0.073	0.078	
	M (0.6-1.2 Mb)	1,280	0.041	0.055	
	L (1.2-1.8 Mb)	2,247	0.005	0.007	
	XL (>3 Mb)	57	0.018	0.018	
Genotyping array	PsychArray	2,728	0.054	0.054	pU; deviance = 24.3, P = 8.3×10^{-7}<br>iIRR; deviance = 6.2, P = 0.013
	GSA	3,841	0.017	0.026	
CNV type	Deletion	2,227	0.050	0.063	pU; deviance = 0.1, P = 0.81<br>iIRR; deviance = 1.0, P = 0.31
	Duplication	4,342	0.023	0.024	

All rCNV calls were visually inspected by at least two analysts and evaluated as; true (T), false (F) or undetermined (U). We derived inter-rater-reliability (IRR) by comparing consensus between raters (T v. F/U). [a]The fraction of calls rated as undetermined (pU) and of calls without consensus between raters during initial rating (iIRR) for bins of increasing LRR-SD, and of each category of the other call properties used to predict pU and iIRR in the initial QC analysis. [b]Calls assigned as (U) and/or (iIRR) were predicted using different call properties; sample LRR-SD, Locus group, Genotyping array, and CNV type (deletion or duplication). Each inspected call was assigned a 0 or 1 for unknown status (U) and a 0 or 1 for lacking initial inter-rater consensus (iIRR). Predictor variables were added sequentially to a logistic model according to significance and significance re-evaluted by comparison with the corresponding nested model with anova.

© 2024 Vaez M et al. JAMA Psychiatry.

eTable4: Prevalence of rCNVs by genotyping array and LRR-SD

Locus	PsychArray		GSA		Array	LRR-SD
	N (carriers)[a]	Prevalence (%) [CI95][b]	N	Prevalence (%) [CI95][b]	P_{FDR} [c]	P_{FDR} [c]
Deletions						
All	921	1.03 [0.919 -1.15]	521	0.941 [0.813 - 1.09]	0.72	0.79
S (<0.6 Mb)	213	0.259 [0.206 - 0.325]	120	0.226 [0.168 - 0.306]	0.79	0.77
M (0.6-1.2 Mb)	186	0.163 [0.123 - 0.216]	106	0.201 [0.146 - 0.277]	0.72	0.77
L (>1.2 Mb)	138	0.157 [0.118 - 0.210]	74	0.127 [0.085 - 0.189]	0.76	0.70
TAR	16	0.032 [0.016 - 0.061]	9	0.024 [0.010 - 0.062]	0.79	0.77
1q21.1	48	0.028 [0.014 - 0.054]	18	0.037 [0.018 - 0.079]	0.79	0.79
2q11.2	12	0.009 [0.003 - 0.029]	5	0.001 [0.001 - 0.001]	0.011	0.79
2q13	35	0.038 [0.021 - 0.068]	22	0.067 [0.038 - 0.117]	0.72	0.55
2q21.1	9	0.008 [0.002 - 0.029]	9	0.013 [0.004 - 0.045]	0.79	0.77
10q11.23	17	0.032 [0.016 - 0.061]	6	0.013 [0.003 - 0.045]	0.72	0.79
13q12.12	12	0.016 [0.007 - 0.041]	5	0.007 [0.001 - 0.037]	0.72	0.77
15q11.2	382	0.438 [0.368 - 0.521]	227	0.399 [0.319 - 0.500]	0.79	0.77
15q13.3	38	0.027 [0.014 - 0.053]	26	0.027 [0.012 - 0.063]	0.99	0.77
16p13.11	79	0.080 [0.053 - 0.120]	50	0.077 [0.046 - 0.129]	0.99	0.65
16p12.1	106	0.124 [0.089 - 0.172]	57	0.118 [0.078 - 0.180]	0.89	0.65
16p11.2d	32	0.041 [0.023 - 0.073]	16	0.031 [0.014 - 0.071]	0.79	0.77
16p11.2	51	0.054 [0.033 - 0.089]	30	0.040 [0.020 - 0.080]	0.79	0.95
17p12	30	0.048 [0.028 - 0.082]	15	0.048 [0.025 - 0.094]	0.99	0.65
17q12	22	0.021 [0.010 - 0.046]	6	0.007 [0.001 - 0.036]	0.72	0.77
22q11.2	20	0.014 [0.005 - 0.035]	16	0.026 [0.011 - 0.062]	0.72	0.77
22q11.2b	12	0.009 [0.003 - 0.029]	11	0.019 [0.007 - 0.054]	0.72	0.77
Duplications						
All	1,257	1.32 [1.19 - 1.45]	718	1.24 [1.09 - 1.41]	0.79	0.77
S (<0.6 Mb)	299	0.306 [0.249 - 0.376]	189	0.336 [0.262 - 0.429]	0.79	0.79
M (0.6-1.2 Mb)	320	0.334 [0.274 - 0.408]	169	0.207 [0.152 - 0.281]	0.17	0.77
L (>1.2 Mb)	247	0.200 [0.155 - 0.257]	137	0.275 [0.209 - 0.362]	0.72	0.77
TAR	105	0.101 [0.071 - 0.145]	70	0.121 [0.080 - 0.182]	0.79	0.89
1q21.1	96	0.089 [0.061 - 0.130]	40	0.030 [0.014 - 0.064]	0.17	0.77
2q13	16	0.028 [0.014 - 0.056]	9	0.007 [0.002 - 0.035]	0.72	0.77
2q21.1	6	0.008 [0.002 - 0.030]	8	0.036 [0.016 - 0.078]	0.58	0.65
10q11.23	5	0.008 [0.002 - 0.030]	8	0.013 [0.004 - 0.045]	0.79	0.77
13q12.12	15	0.017 [0.007 - 0.041]	8	0.007 [0.001 - 0.035]	0.72	0.79
15q11.2	361	0.473 [0.400 - 0.560]	218	0.415 [0.332 - 0.518]	0.72	0.55
PWAS	38	0.012 [0.005 - 0.029]	11	0.013 [0.004 - 0.045]	0.99	0.77
15q13.3	69	0.060 [0.038 - 0.095]	35	0.086 [0.052 - 0.141]	0.72	0.55
16p13.11	185	0.185 [0.142 - 0.241]	109	0.156 [0.109 - 0.223]	0.79	0.79
16p12.1	27	0.040 [0.023 - 0.072]	20	0.049 [0.026 - 0.095]	0.79	0.81
16p11.2d	44	0.046 [0.027 - 0.079]	24	0.044 [0.022 - 0.087]	0.99	0.79
16p11.2	118	0.114 [0.081 - 0.160]	67	0.086 [0.053 - 0.138]	0.72	0.65
17p12	18	0.024 [0.012 - 0.051]	6	0.024 [0.009 - 0.062]	0.99	0.77
17q12	36	0.023 [0.011 - 0.047]	29	0.045 [0.023 - 0.088]	0.72	0.65
22q11.2	104	0.068 [0.044 - 0.104]	52	0.106 [0.068 - 0.165]	0.72	0.55
22q11.2b	19	0.025 [0.012 - 0.051]	10	0.007 [0.002 - 0.034]	0.72	0.55

[a]Total number of rCNV carriers of each class and locus genotyped on each array (out of a total of 73,857 and 46,390 individuals genotyped on the Illumina PsychArray and GSA, respectively). [b]Population-based CNV prevalence (with 95% CI) for each class, calculated using finite population correction weights with the svydesign() and svyciprop() functions from the survey package in R. [c]The last two columns detail the P-value from logistic regression models predicting carrier status for each respective CNV class from genotyping array and sample LRR-SD, respectively, using the svyglm() function from the survey package in R; the P-values were adjusted for false discovery rate using the p.adjust (method="fdr") function in R.

© 2024 Vaez M et al. JAMA Psychiatry.

eTable5: LRR-SD, case- and QC-fractions, and rCNV prevalence by genotyping wave

Genotyping wave	N (total)	LRR-SD (mean)	Case (%)	QC (%)
iPSYCH2012 (Psych Array)				
w1	3,787	0.185	68.6	1.2
w2	5,531	0.192	45.1	1.2
w3	3,124	0.199	62.9	0.8
w4	3,022	0.190	61.5	1.2
w5	2,829	0.213	61.9	1.2
w6	2,834	0.226	61.9	1.7
w7	2,710	0.227	64.1	2.9
w8	3,342	0.226	65.2	0.5
w9	3,564	0.214	68.1	0.3
w10	2,686	0.220	71.3	0.7
w11	2,813	0.236	71.6	1.5
w12	2,668	0.239	71.0	1.0
w13	2,659	0.233	70.7	1.1

Genotyping wave	N (total)	LRR-SD (mean)	Case (%)	QC (%)
w14	2,669	0.240	71.3	2.0
w15	3,073	0.215	71.3	0.6
w16	3,074	0.227	71.2	1.7
w17	2,987	0.231	71.6	2.0
w18	3,337	0.192	72.0	1.1
w19	3,334	0.205	72.2	3.9
w20	2,992	0.213	71.5	1.8
w21	3,369	0.211	72.2	0.9
w22	3,328	0.221	71.9	1.6
w23	4,183	0.234	75.7	8.2
w24	294	0.193	87.1	<1.7
w25	1,013	0.174	3.8	4.9
iPSYCH2015i (Global Screening Array)				
w26	48,155	0.186	65.2	3.7

iPSYCH2012 samples were genotyped in 25 consecutive batches (waves), while iPSCYH2015i samples were genotyped in one batch. As definition of genotyping clusters in each batch can affect the LRR and BAF values we compared mean sample LRR-SD, as well as the proportion of case samples and samples lost to QC in the CNV analysis, across batches. Waves 23 and 25 differ from other batches through high proportion of samples lost to QC and low proportion of case samples, respectively. Consequently, we compared the population-based prevalence of deletions and duplications in those two batches against all other iPSYCH2012 waves, but found no indication of significant differences (pDel = 1.12% and 1.29% vs 1.01%; P = 0.69 and 0.49; and pDup = 1.10% and 1.18% vs 1.01%; P = 0.40 and 0.70; for w23 and w25 vs other iPSYCH2012 waves, respectively).

eTable6: iPSYCH2015 samples by diagnosis group and genotyping array

Diagnosis[a]	PsychArray	GSA	Total
Cohort	26,783	16,528	43,311
ASD	14,943	7,224	22,167
ADHD	17,407	8,779	26,186
SSD	5,711	7,415	13,126
MDD	21,076	10,546	31,622

Diagnosis[b]	PsychArray	GSA	Total
SCZ	3,205	3,399	6,604
BPD	1,825	1,185	3,010
AFF	22,411	11,597	34,008
EPI	2,764	1,240	4,004
ID	4,295	1,713	6,008

iPSYCH2012 samples were genotyped on Illumina PsychArray whereas iPSYCH2015i samples were genotyped on Illumina Global Screening Array. Cohort; population subcohort individuals, ASD; autism spectrum disorder, ADHD; attention-deficit hyperactivity disorder, SSD; schizophrenia spectrum disorder, MDD; major depressive disorder, SCZ; schizophrenia (ICD10: F20), BPD; bipolar disorder, AFF; any affective disorder, EPI; epilepsy, ID; intellectual disability (EPI and ID were not target disorders in the iPSYCH2015 case-cohort design).

© 2024 Vaez M et al. JAMA Psychiatry.

eTable7: rCNV prevalence in iPSYCH2015 and the UKB

	Deletion prevalence				Duplication prevalence				
**rCNV locus**	**iPSYCH (n)[a]**	**iPSYCH (%) [CI95%][b]**	**UKB (%) [CI95%][c]**	P_{FDR}[d]	**iPSYCH (n)[a]**	**iPSYCH (%) [CI95%][b]**	**UKB (%) [CI95%][c]**	P_{FDR}[d]	**Del-v-dup P_{FDR}[e]**
TAR	25	0.029 [0.017-0.049]	0.018 [0.016-0.020]	0.18	175	0.109 [0.083-0.143]	0.103 [0.098-0.108]	0.74	0.00025
1q21.1	66	0.032 [0.019-0.052]	0.027 [0.024-0.029]	0.62	136	0.066 [0.047-0.093]	0.042 [0.039-0.045]	0.081	0.041
2q11.2	17	0.006 [0.002-0.017]	0.007 [0.006-0.009]	0.71	6	0.009 [0.004-0.024]	0.007 [0.006-0.008]	0.72	0.61
2q13	57	0.049 [0.033-0.074]	0.013 [0.011-0.015]	1.0×10^{-6}	25	0.020 [0.010-0.038]	0.017 [0.015-0.019]	0.74	0.043
2q21.1	18	0.010 [0.004-0.025]	0.010 [0.008-0.011]	0.90	14	0.019 [0.010-0.037]	0.014 [0.012-0.016]	0.62	0.42
10q11.23	23	0.024 [0.013-0.043]	0.014 [0.012-0.015]	0.14	13	0.010 [0.004-0.025]	0.010 [0.009-0.012]	0.95	0.18
13q12.12	17	0.012 [0.006-0.028]	0.020 [0.018-0.023]	0.36	23	0.013 [0.006-0.028]	0.056 [0.053-0.060]	0.0023	0.94
15q11.2	609	0.423 [0.368-0.485]	0.395 [0.385-0.405]	0.48	579	0.450 [0.393-0.515]	0.484 [0.473-0.495]	0.58	0.61
PWAS	5	0.009 [0.004-0.024]	na.	na.	49	0.012 [0.006-0.026]	0.005 [0.004-0.006]	0.081	0.68
15q13.3	64	0.027 [0.016-0.046]	0.010 [0.009-0.012]	0.0037	104	0.070 [0.050-0.099]	0.059 [0.056-0.063]	0.58	0.0078
16p13.11	129	0.079 [0.057-0.108]	0.031 [0.029-0.034]	2.1×10^{-6}	294	0.174 [0.140-0.215]	0.197 [0.190-0.204]	0.58	0.00028
16p12.1	163	0.122 [0.094-0.157]	0.058 [0.055-0.062]	2.1×10^{-6}	47	0.044 [0.028-0.068]	0.048 [0.045-0.052]	0.74	0.00031
16p11.2d	48	0.037 [0.023-0.059]	0.014 [0.012-0.016]	7.5×10^{-4}	68	0.045 [0.030-0.069]	0.033 [0.030-0.035]	0.51	0.61
16p11.2	81	0.048 [0.032-0.073]	0.026 [0.024-0.029]	0.013	185	0.103 [0.078-0.136]	0.033 [0.030-0.036]	1.1×10^{-10}	0.0078
17p12	45	0.048 [0.032-0.073]	0.056 [0.053-0.060]	0.59	24	0.024 [0.013-0.044]	0.029 [0.027-0.032]	0.72	0.12
17q12	28	0.016 [0.008-0.032]	0.002 [0.002-0.003]	1.9×10^{-4}	65	0.032 [0.019-0.052]	0.024 [0.022-0.027]	0.58	0.18
22q11.2	36	0.018 [0.010-0.035]	0.002 [0.002-0.003]	3.3×10^{-5}	156	0.083 [0.061-0.113]	0.067 [0.063-0.071]	0.54	0.00028
22q11.2b	23	0.013 [0.006-0.028]	na.	na.	29	0.018 [0.009-0.035]	na.	na.	0.61

[a]Total number of deletion and duplication carriers in iPSYCH2015 at the 18 herein studied rCNV loci. [b]Population-based prevalence and CI95% were calculated with svydesign() and svyciprop() functions from the survey package in R using finite population correction (fpc) to account for oversampling of cases. [c]rCNV prevalence in the UKB was calculated based on published carrier counts[7] [d]Differences in rCNV prevalence were assessed with a Welch's test, and P-values adjusted for multiple comparisons with the false-discovery-rate (FDR) option of the p.adjust function in the stats package in R between iPSYCH2015 and UKB, and [e]between deletions and duplications at each locus in iPSYCH2015.

© 2024 Vaez M et al. JAMA Psychiatry.

eTable8: rCNV-associated risk of psychiatric disorders in iPSYCH2015

Locus	Attention-deficit hyperactivity disorder (ADHD)				Autism spectrum disorder (ASD)			
	Deletion		Duplication		Deletion		Duplication	
	HR [CI95%][a]	P	HR [CI95%][a]	P	HR [CI95%][a]	P	HR [CI95%][a]	P
TAR	0.59 [0.18-1.87]	0.37	1.76 [1.15-2.69]	0.0086	0.64 [0.20-2.03]	0.44	1.49 [0.95-2.34]	0.084
1q21.1	1.93 [0.92-4.07]	0.083	2.65 [1.56-4.49]	2.9E-04	2.37 [1.16-4.86]	0.018	4.03 [2.44-6.64]	4.6E-08
2q11.2	3.42 [0.88-13.2]	0.075	na.[b]	na.[b]	2.44 [0.54-11.0]	0.25	na.[b]	na.[b]
2q13	1.68 [0.83-3.36]	0.15	1.43 [0.49-4.21]	0.51	0.92 [0.40-2.08]	0.84	0.97 [0.27-3.45]	0.97
2q21.1	2.71 [0.69-10.6]	0.15	na.[b]	na.[b]	2.49 [0.64-9.80]	0.19	0.61 [0.12-3.14]	0.55
10q11.23	0.32 [0.07-1.52]	0.15	na.[b]	na.[b]	0.74 [0.22-2.51]	0.63	1.81 [0.35-9.35]	0.48
13q12.12	2.34 [0.82-6.68]	0.11	2.65 [0.67-10.4]	0.16	0.77 [0.15-3.98]	0.75	5.00 [1.47-17.0]	0.010
15q11.2	1.43 [1.14-1.80]	0.0021	1.42 [1.13-1.79]	0.0026	1.45 [1.15-1.84]	0.0018	1.07 [0.82-1.38]	0.63
PWAS	na.[b]	na.[b]	3.64 [1.04-12.8]	0.044	na.[b]	na.[b]	20.8 [7.86-55.0]	9.6E-10
15q13.3	2.51 [1.09-5.78]	0.031	1.77 [0.98-3.19]	0.057	3.74 [1.66-8.42]	0.0014	1.59 [0.85-2.98]	0.15
16p13.11	2.26 [1.37-3.73]	0.0014	2.01 [1.43-2.81]	5.0E-05	1.92 [1.12-3.31]	0.018	1.84 [1.30-2.61]	5.9E-04
16p12.1	1.86 [1.22-2.84]	0.0039	0.56 [0.24-1.33]	0.19	1.11 [0.68-1.83]	0.68	1.00 [0.46-2.19]	1.00
16p11.2d	1.26 [0.55-2.88]	0.58	1.57 [0.78-3.18]	0.21	0.94 [0.37-2.39]	0.89	1.26 [0.57-2.79]	0.57
16p11.2	0.77 [0.36-1.61]	0.48	2.63 [1.75-3.95]	3.3E-06	3.07 [1.75-5.36]	8.4E-05	2.04 [1.33-3.15]	0.0012
17p12	0.45 [0.18-1.15]	0.095	0.69 [0.21-2.23]	0.53	0.81 [0.36-1.82]	0.61	0.64 [0.17-2.41]	0.51
17q12	4.24 [1.29-13.9]	0.017	4.19 [1.98-8.87]	1.8E-04	7.79 [2.71-22.4]	1.4E-04	3.12 [1.37-7.08]	0.0065
22q11.2	1.17 [0.49-2.80]	0.72	2.61 [1.67-4.09]	2.6E-05	1.50 [0.59-3.84]	0.39	2.63 [1.66-4.16]	3.4E-05
22q11.2b	1.81 [0.53-6.18]	0.35	1.63 [0.55-4.83]	0.38	2.34 [0.67-8.13]	0.18	0.92 [0.25-3.30]	0.89

Locus	Major depressive disorder (MDD)				Schizophrenia spectrum disorder (SSD)			
TAR	0.96 [0.28-3.28]	0.95	1.45 [0.92-2.31]	0.11	1.63 [0.45-5.83]	0.45	1.02 [0.56-1.86]	0.95
1q21.1	2.08 [0.86-5.04]	0.10	1.43 [0.74-2.75]	0.28	1.78 [0.68-4.69]	0.24	2.7 [1.37-5.33]	0.0042
2q11.2	2.18 [0.61-7.86]	0.23	na.[b]	na.[b]	4.75 [1.07-21.1]	0.041	na.[b]	na.[b]
2q13	1.39 [0.60-3.22]	0.44	0.79 [0.20-3.04]	0.73	1.58 [0.59-4.19]	0.36	1.23 [0.29-5.18]	0.78
2q21.1	na.[b]	na.[b]	0.52 [0.12-2.29]	0.39	2.5 [0.28-22.8]	0.42	na.[b]	na.[b]
10q11.23	0.77 [0.22-2.68]	0.69	1.00 [0.18-5.66]	1.00	0.66 [0.13-3.3]	0.61	3.22 [0.60-17.1]	0.17
13q12.12	0.39 [0.11-1.42]	0.15	2.67 [0.56-12.8]	0.22	na.[b]	na.[b]	na.[b]	na.[b]
15q11.2	1.17 [0.91-1.52]	0.22	1.07 [0.82-1.38]	0.62	0.99 [0.71-1.36]	0.93	1.32 [0.97-1.78]	0.077
PWAS	na.[b]	na.[b]	1.84 [0.31-10.9]	0.50	na.[b]	na.[b]	9.19 [1.72-49.2]	0.010
15q13.3	2.16 [0.84-5.50]	0.11	1.54 [0.86-2.78]	0.15	3.49 [1.36-8.95]	0.0093	1.44 [0.71-2.95]	0.31
16p13.11	1.06 [0.56-2.03]	0.85	1.38 [0.94-2.01]	0.10	2.29 [1.22-4.30]	0.010	1.98 [1.31-3.00]	0.0012
16p12.1	0.91 [0.54-1.52]	0.71	0.86 [0.40-1.87]	0.71	1.21 [0.66-2.20]	0.54	0.48 [0.14-1.68]	0.25
16p11.2d	1.04 [0.40-2.73]	0.94	1.17 [0.56-2.42]	0.67	2.41 [0.96-6.08]	0.062	1.81 [0.81-4.04]	0.15
16p11.2	0.42 [0.18-1.00]	0.049	1.04 [0.63-1.74]	0.87	1.26 [0.55-2.88]	0.58	1.50 [0.89-2.54]	0.13
17p12	0.85 [0.38-1.91]	0.70	0.55 [0.18-1.71]	0.30	0.46 [0.13-1.65]	0.24	1.17 [0.34-3.99]	0.80
17q12	na.[b]	na.[b]	1.47 [0.53-4.07]	0.46	4.84 [0.81-28.9]	0.084	3.19 [1.17-8.68]	0.023
22q11.2	1.03 [0.30-3.52]	0.96	1.02 [0.59-1.77]	0.94	3.28 [1.15-9.30]	0.026	1.34 [0.70-2.54]	0.37
22q11.2b	1.11 [0.25-4.81]	0.89	1.32 [0.43-4.08]	0.63	2.60 [0.65-10.4]	0.18	2.51 [0.77-8.16]	0.12

Table continues on next page

© 2024 Vaez M et al. JAMA Psychiatry.

Locus	Any iPSYCH2015 disorder (ANY)				Any affective disorder (AFF)			
	Deletion		Duplication		Deletion		Duplication	
	HR [CI95%][a]	P	HR [CI95%][a]	P	HR [CI95%][a]	P	HR [CI95%][a]	P
TAR	0.68 [0.29-1.60]	0.38	1.48 [1.02-2.14]	0.037	0.90 [0.26-3.06]	0.86	1.37 [0.87-2.18]	0.18
1q21.1	2.12 [1.08-4.14]	0.028	2.54 [1.58-4.08]	0.0001	2.13 [0.89-5.09]	0.087	1.45 [0.76-2.77]	0.25
2q11.2	2.76 [0.93-8.12]	0.067	0.31 [0.05-1.90]	0.21	2.06 [0.57-7.43]	0.27	na.[b]	na.[b]
2q13	1.28 [0.69-2.38]	0.43	1.10 [0.43-2.82]	0.85	1.29 [0.56-2.99]	0.56	0.73 [0.19-2.82]	0.65
2q21.1	2.34 [0.61-8.87]	0.21	0.45 [0.14-1.45]	0.18	1.51 [0.17-13.2]	0.71	0.65 [0.17-2.53]	0.53
10q11.23	0.71 [0.28-1.78]	0.47	1.63 [0.44-6.05]	0.46	0.72 [0.21-2.48]	0.60	1.40 [0.30-6.57]	0.67
13q12.12	0.71 [0.26-1.91]	0.50	3.81 [1.25-11.6]	0.019	0.36 [0.10-1.32]	0.12	3.03 [0.66-13.9]	0.15
15q11.2	1.30 [1.07-1.58]	0.0085	1.21 [1.00-1.47]	0.053	1.15 [0.89-1.49]	0.28	1.09 [0.84-1.40]	0.51
PWAS	na.[b]	na.[b]	11.3 [4.24-30.1]	1.3E-06	na.[b]	na.[b]	1.70 [0.29-10.1]	0.56
15q13.3	2.70 [1.31-5.56]	0.0070	1.53 [0.94-2.50]	0.091	2.01 [0.79-5.13]	0.14	1.48 [0.82-2.66]	0.19
16p13.11	1.75 [1.11-2.76]	0.017	1.70 [1.27-2.27]	0.0003	1.14 [0.61-2.14]	0.69	1.42 [0.97-2.06]	0.069
16p12.1	1.26 [0.86-1.84]	0.24	0.81 [0.45-1.44]	0.47	0.99 [0.60-1.62]	0.96	0.87 [0.41-1.85]	0.71
16p11.2d	1.23 [0.62-2.44]	0.56	1.32 [0.73-2.40]	0.36	0.97 [0.37-2.54]	0.95	1.20 [0.58-2.45]	0.63
16p11.2	na.[b]	na.[b]	na.[b]	na.[b]	0.45 [0.20-1.03]	0.057	0.99 [0.60-1.65]	0.98
17p12	0.61 [0.33-1.15]	0.13	0.64 [0.27-1.51]	0.31	0.79 [0.35-1.77]	0.57	0.52 [0.17-1.59]	0.25
17q12	na.[b]	na.[b]	na.[b]	na.[b]	0.63 [0.08-5.11]	0.68	1.56 [0.57-4.26]	0.38
22q11.2	1.85 [0.83-4.14]	0.13	1.85 [1.22-2.80]	0.0035	0.95 [0.28-3.24]	0.93	0.98 [0.57-1.70]	0.95
22q11.2b	1.64 [0.56-4.82]	0.37	1.58 [0.64-3.89]	0.32	1.03 [0.24-4.49]	0.97	1.84 [0.66-5.16]	0.25

Locus	Bipolar disorder (BPD)				Schizophrenia (SCZ)			
TAR	na.[b]	na.[b]	na.[b]	na.[b]	na.[b]	na.[b]	1.50 [0.77-2.93]	0.23
1q21.1	3.52 [0.88-14.1]	0.076	1.63 [0.37-7.32]	0.52	1.75 [0.52-5.84]	0.36	2.36 [0.97-5.71]	0.058
2q11.2	na.[b]	na.[b]	na.[b]	na.[b]	7.77 [1.25-48.2]	0.028	na.[b]	na.[b]
2q13	na.[b]	na.[b]	na.[b]	na.[b]	1.39 [0.37-5.18]	0.63	1.63 [0.31-8.59]	0.57
2q21.1	na.[b]	na.[b]	na.[b]	na.[b]	na.[b]	na.[b]	na.[b]	na.[b]
10q11.23	na.[b]	na.[b]	na.[b]	na.[b]	1.3 [0.25-6.66]	0.76	na.[b]	na.[b]
13q12.12	na.[b]	na.[b]	na.[b]	na.[b]	na.[b]	na.[b]	12.7 [2.17-74.1]	0.0048
15q11.2	0.63 [0.30-1.31]	0.22	1.26 [0.72-2.20]	0.41	1.29 [0.88-1.88]	0.19	1.25 [0.84-1.87]	0.27
PWAS	na.[b]	na.[b]	na.[b]	na.[b]	na.[b]	na.[b]	6.88 [0.95-49.8]	0.056
15q13.3	na.[b]	na.[b]	0.85 [0.19-3.79]	0.84	3.28 [1.02-10.6]	0.047	1.30 [0.50-3.37]	0.59
16p13.11	2.83 [1.11-7.23]	0.029	0.80 [0.28-2.27]	0.67	1.77 [0.78-4.01]	0.17	2.05 [1.22-3.45]	0.0069
16p12.1	1.62 [0.65-4.03]	0.30	na.[b]	na.[b]	1.34 [0.64-2.8]	0.44	0.65 [0.15-2.87]	0.56
16p11.2d	na.[b]	na.[b]	1.89 [0.51-6.93]	0.34	2.42 [0.78-7.52]	0.13	2.38 [0.95-5.91]	0.063
16p11.2	na.[b]	na.[b]	0.53 [0.13-2.28]	0.40	0.57 [0.13-2.5]	0.45	1.22 [0.59-2.52]	0.58
17p12	na.[b]	na.[b]	na.[b]	na.[b]	na.[b]	na.[b]	2.38 [0.69-8.20]	0.17
17q12	na.[b]	na.[b]	2.15 [0.39-11.8]	0.38	5.38 [0.61-47.2]	0.13	3.25 [0.98-10.7]	0.054
22q11.2	na.[b]	na.[b]	na.[b]	na.[b]	3.18 [0.92-10.9]	0.067	2.02 [0.99-4.12]	0.053
22q11.2b	na.[b]	na.[b]	na.[b]	na.[b]	na.[b]	na.[b]	2.56 [0.59-11.0]	0.21

[a]Population-valid rCNV-associated hazard ratios (HR) and 95% confidence intervals (CI95%) were derived for each psychiatric disorder targeted in the iPSYCH2015 case-cohort design, using a Cox proportional hazards (CPH) model with inverse probability of sampling weights.
[b]Comparisons involving <2 case carriers, or the CPH model failing a test of proportionality of hazards, were excluded.

© 2024 Vaez M et al. JAMA Psychiatry.

eTable9: Comparison of risk estimates in iPSYCH2015 with case-control studies

ADHD (case-control study; Gudmundsson *et al.*[20])

rCNV	HR [CI95%][a]	OR [CI95%][b]	P_{FDR}[c]
15q11.2_del	1.43 [1.14-1.80]	1.65 [1.11-2.37]	0.72
15q13.3_del	2.51 [1.09-5.78]	5.97[2.63-12.6]	0.46
16p11.2_del	0.77 [0.36-1.61]	2.16 [0.75-5.11]	0.41
16p11.2_dup	2.63 [1.75-3.95]	4.34 [2.27-7.81]	0.47
16p11.2d_del	1.26 [0.56-2.88]	2.19[0.42-7.29]	0.72
16p12.1_del	1.86 [1.22-2.85]	1.52 [0.63-3.16]	0.72
16p13.11_dup	2.00 [1.43-2.81]	2.12 [1.31-3.27]	0.85
17p12_del	0.45 [0.18-1.15]	2.20 [0.67-5.66]	0.19
17q12_dup	4.19 [1.98-8.87]	2.20 [0.76-5.24]	0.65
1q21.1_del	1.93 [0.92-4.07]	2.68 [0.92-6.44]	0.72
1q21.1_dup	2.65 [1.56-4.49]	3.44 [1.67-6.52]	0.72
22q11.2_del	1.17 [0.49-2.80]	10.7 [4.66-23.2]	0.0032
22q11.2_dup	2.61 [1.67-4.09]	2.24 [1.32-3.63]	0.72

MDD (case-control study; Kendall *et al.*[21])

rCNV	HR [CI95%][a]	OR [CI95%][b]	P_{FDR}[c]
15q11.2_del	1.17 [0.91-1.52]	1.11 [0.90-1.35]	0.87
15q13.3_del	2.15 [0.84-5.50]	0.77 [0.13-2.52]	0.56
16p11.2_del	0.42 [0.18-1.00]	1.2 1[0.54-2.34]	0.49
16p11.2_dup	1.04[0.63-1.74]	2.65 [1.53-4.31]	0.22
16p11.2d_del	1.04 [0.40-2.73]	2.23 [0.92-4.63]	0.56
16p11.2d_dup	1.17 [0.56-2.42]	1.57 [0.82-2.73]	0.79
16p12.1_del	0.91 [0.54-1.52]	1.47 [0.90-2.27]	0.49
16p13.11_del	1.06 [0.56-2.02]	2.21 [1.25-3.63]	0.49
16p13.11_dup	1.38 [0.94-2.01]	0.87 [0.63-1.78]	0.49
17q12_dup	1.47 [0.53-4.07]	1.55 [0.69-3.02]	0.98
1q21.1_del	2.08 [0.86-5.04]	1.11 [0.46-2.22]	0.56
1q21.1_dup	1.43 [0.74-2.75]	2.17 [1.34-3.36]	0.56
22q11.2_del	1.03 [0.30-3.52]	1.69 [0.09-9.17]	0.87
22q11.2_dup	1.02 [0.59-1.77]	1.72 [1.12-2.53]	0.49
2q11.2_del	2.18[0.60-7.86]	2.34 [0.69-6.02]	0.98
2q13_del	1.39 [0.60-3.22]	0.98 [0.24-2.67]	0.85
2q13_dup	0.79 [0.20-3.04]	1.29 [0.49-2.90]	0.79
PWAS_dup	1.84 [0.31-10.9]	8.14 [2.77-21.7]	0.49
TAR_del	0.96 [0.28-3.28]	0.95 [0.29-2.29]	0.99
TAR_dup	1.45 [0.92-2.31]	1.17 [0.77-1.69]	0.79

ASD (case-control study; Malhotra *et al.*[19])

rCNV	HR [CI95%][a]	OR [CI95%][b]	P_{FDR}[c]
15q11.2_del	1.45 [1.15-1.84]	0.30 [0.10-1.40]	0.081
15q13.3_del	3.74 [1.66-8.42]	10.8 [3.50-33.1]	0.29
16p11.2_del	3.06 [1.75-5.36]	9.50 [5.20-17.4]	0.039
16p11.2_dup	2.04 [1.33-3.15]	11.8 [6.10-22.7]	0.00014
16p13.11_dup	1.84 [1.30-2.61]	1.50 [0.50-4.00]	0.72
17p12_del	0.81 [0.36-1.82]	4.00 [0.90-17.5]	0.18
17q12_del	7.79 [2.71-22.4]	16.0 [2.90-87.9]	0.66
1q21.1_del	2.37 [1.16-4.86]	1.60 [0.20-11.7]	0.72
1q21.1_dup	4.03 [2.44-6.64]	8.00 [3.50-18.4]	0.31
22q11.2_dup	2.63 [1.67-4.16]	3.30 [1.60-6.60]	0.72
PWAS_dup	20.8 [7.86-55.0]	42.6 [15.7-115]	0.49

SSD (case-control studies; Marshall *et al.*[22], Rees *et al.*[23,24])

rCNV	HR [CI95%][a]	OR [CI95%][b]	P_{FDR}[c]
15q11.2_del[24]	0.98 [0.71-1.36]	2.15 [1.71-2.68]	0.0019
15q13.3_del[24]	3.49 [1.36-8.95]	7.52 [3.98-14.2]	0.44
PWAS_dup[24]	9.19 [1.72-49.2]	13.2 [3.72-46.8]	0.78
16p11.2d_del[24]	2.41 [0.96-6.08]	3.39 [1.21-9.52]	0.78
16p13.11_dup[24]	1.98 [1.31-3.00]	2.30 [1.57-3.36]	0.78
17p12_del[24]	0.46 [0.13-1.65]	3.62 [1.73-7.57]	0.029
17q12_del[24]	4.84 [0.81-28.8]	6.64 [1.78-24.7]	0.78
1q21.1_del[24]	1.78 [0.68-4.69]	8.35 [4.65-15.0]	0.029
1q21.1_dup[24]	2.70 [1.37-5.33]	3.45 [1.92-6.20]	0.78
TAR_del[23]	1.63[0.45-5.83]	1.20 [0.36-3.90]	0.78
TAR_dup[23]	1.02 [0.56-1.86]	1.90 [0.93-3.93]	0.44
2q11.2_del[23]	4.75 [1.07-21.1]	9.30 [1.03-447]	0.78
2q13_del[23]	1.58 [0.59-4.18]	3.60 [0.26-206]	0.78
2q13_dup[23]	1.23 [0.29-5.18]	1.70 [0.30-9.98]	0.78
16p11.2d_dup[23]	1.81 [0.81-4.04]	1.20 [0.44-3.08]	0.78
16p11.2_del[23]	1.26 [0.55-2.88]	0.62 [0.19-1.79]	0.64
16p12.1_del[23]	1.21 [0.66-2.20]	3.30 [1.61-7.05]	0.12
16p13.11_del[23]	2.29 [1.22-4.30]	1.10 [0.43-2.73]	0.44
17q12_dup[23]	3.19 [1.17-8.68]	2.20 [0.74-6.76]	0.78
16p11.2_dup[22]	1.50 [0.89-2.54]	9.40 [4.20-20.9]	0.0019
22q11.2_del[22]	3.28 [1.15-9.30]	67.7 [9.40-492]	0.029
22q11.2_dup[22]	1.34 [0.70-2.54]	0.15 [0.04-0.52]	0.021

[a]rCNV-associated HRs and 95% confidence intervals (CI95%) for ASD, ADHD, MDD, and SSD in iPSYCH2015 for those rCNVs with available risk estimates in case-control studies of the corresponding disorder. [b] Odds ratios (ORs) and 95% confidence intervals (CI95%) of rCNV-associated risk of ASD, ADHD, MDD, and SSD reported in published case-control studies. [C]Difference between HRs in iPSYCH2015 and previously reported ORs was evaluated with a Welch's t-test and P-values adjusted for multiple comparisons for each disorder separately with a false discovery rate (FDR) test using the p.adjust() function from the stat package in R. Note that the diagnostic criteria for specific disorders may differ between iPSYCH2015 and the respective comparison case-control study.

© 2024 Vaez M et al. JAMA Psychiatry.

eTable10: Sensitivity analysis of rCNV-associated risk of iPSYCH2015 outcomes

Comparison of Hazard ratios (HRs) with Odds ratios (ORs) associated with rCNVs across iPSYCH2015 psychiatric outcomes. For sensitivity analysis, we computed rCNV-associated ORs with 95% confidence intervals (CI95%) using generalised linear models (GLMs) to compare with weighted Cox proportional HRs (see Method).

This table is provided in a separate supplementary file (eTable10.pdf)

eTable11: Diagnosis-specific rCNV effects on risk between iPSYCH2015 disorders

Locus	rCNV	ASD v. ADHD[a]	P_{FDR}[b]	SSD v. ADHD[a]	P_{FDR}[b]	SSD v. ASD[a]	P_{FDR}[b]
TAR	del	-0.27	0.8363	1.28	0.3115	1.55	0.0324
	dup	-0.35	0.3902	-0.79	0.120	-0.44	0.4877
1q21.1	del	0.24	0.7626	-0.39	0.7065	-0.65	0.5233
	dup	0.75	0.0821	-0.32	0.6001	-1.04	0.0330
2q11.2	del	-0.60	0.7226	0.53	0.7077	1.12	0.5822
	dup	na.[c]	na.[c]	na.[c]	na.[c]	na.[c]	na.[c]
2q13	del	-0.69	0.2962	-0.29	0.7077	0.35	0.7802
	dup	-0.59	0.7210	-0.31	0.7802	0.29	0.9148
2q21.1	del	-0.03	0.9753	-1.08	0.4956	-0.95	0.6774
	dup	na.[c]	na.[c]	na.[c]	na.[c]	na.[c]	na.[c]
10q11.23	del	1.90	0.2962	1.69	0.4383	-0.18	0.9549
	dup	na.[c]	na.[c]	na.[c]	na.[c]	0.68	0.7802
13q12.12	del	-1.98	0.0593	na.[c]	na.[c]	na.[c]	na.[c]
	dup	1.14	0.3486	na.	na.[c]	na.[c]	na.[c]
15q11.2	del	0.02	0.9021	-0.53	0.0327	-0.56	0.0252
	dup	-0.43	0.0495	-0.17	0.5873	0.25	0.4559
PWAS	del	na.[c]	na.[c]	na.[c]	na.[c]	na.[c]	na.[c]
	dup	3.53	2.2×10^{-16}	0.71	0.5836	-2.45	0.0013
15q13.3	del	0.74	0.3077	0.31	0.7077	-0.39	0.7595
	dup	-0.24	0.7226	-0.08	0.8764	0.15	0.8990
16p13.11	del	-0.40	0.4148	-0.13	0.7936	0.24	0.7786
	dup	-0.13	0.7226	-0.13	0.7077	0.00	0.9979
16p12.1	del	-0.83	0.0445	-0.61	0.2321	0.19	0.8124
	dup	0.92	0.3326	0.09	0.9135	-0.81	0.4877
16p11.2d	del	-0.28	0.780	0.91	0.3867	1.18	0.2432
	dup	-0.51	0.5194	0.31	0.7077	0.80	0.4244
16p11.2	del	2.17	0.0001	0.64	0.4956	-1.42	0.0282
	dup	-0.57	0.1171	-0.88	0.0327	-0.38	0.4973
17p12	del	0.78	0.3077	-0.35	0.7802	-1.11	0.4544
	dup	-0.14	0.9021	0.91	0.5524	1.05	0.4544
17q12	del	1.10	0.2860	-0.15	0.8764	-1.11	0.4345
	dup	-0.65	0.3411	-0.64	0.4882	-0.05	0.9549
22q11.2	del	0.43	0.7226	1.26	0.2321	0.84	0.4639
	dup	-0.14	0.780	-1.05	0.0227	-0.91	0.0788
22q11.2b	del	0.24	0.8377	0.63	0.6731	0.39	0.8124
	dup	-0.83	0.4999	0.47	0.7077	1.26	0.4244

[a]Coefficients of the interaction term between diagnosis and rCNV were extracted from the results of GEE models corresponding to three pairwise comparisons across ASD, ADHD, and SSD using glmgee() function from the glmtoolbox package in R (see eMethods). [b]P-values corresponding to each coefficient of the interaction term between diagnosis and rCNV within each pairwise diagnosis were retrieved from the GEE models and adjusted for multiple comparisons with the false-discovery-rate (FDR) by p.adjust() function from the stat package in R. [c]We included only those rCNVs in each GEE analysis that had valid HR estimates for both diagnoses within each pairwise comparison.

© 2024 Vaez M et al. JAMA Psychiatry.

eTable12: Gene constraint at rCNV loci

The table details gene constraint data from the GnomAD database (v2.1.1)[17] for all genes that are overlapped at least 50% by rCNV loci in our study. The loss-of-function observed/expected upper fraction (LOEUF) scores in GnomAD are constructed such that lower scores indicate more gene constraint (i.e. intolerance to loss-of-function mutations); therefore, we first inverted the scores, and then derived the sum of the inverted scores (1/LOEUF) for all genes at each rCNV locus.

This table is provided in a separate supplementary file (eTable12.pdf)

eTable13: rCNV-associated risk of other brain disorders in iPSYCH2015

	Deletion		Duplication		Deletion		Duplication	
	**HR [CI95%][a]**	**P**	**HR [CI95%][a]**	**P**	**HR [CI95%][a]**	**P**	**HR [CI95%][a]**	**P**
**Locus**	**Intellectual disability (ID)**				**Epilepsy (EPI)**			
TAR	1.18 [0.26-5.29]	0.83	1.18 [0.55-2.51]	0.67	na.[b]	na.[b]	1.49 [0.65-3.42]	0.34
1q21.1	5.38 [2.40-12.1]	4.6×10^{-5}	3.14 [1.59-6.20]	9.9×10^{-4}	1.63 [0.41-6.47]	0.49	1.79 [0.66-4.88]	0.25
2q11.2	13.4 [2.97-60.7]	7.4×10^{-4}	na.[b]	na.[b]	9.52 [2.42-37.4]	0.0013	na.[b]	na.[b]
2q13	1.54 [0.52-4.56]	0.44	0.83 [0.10-6.68]	0.86	0.64 [0.09-4.69]	0.66	na.[b]	na.[b]
2q21.1	4.67 [1.27-17.2]	0.021	2.05 [0.41-10.1]	0.38	4.17 [0.48-36.1]	0.20	na.[b]	na.[b]
10q11.23	0.67 [0.08-5.29]	0.70	1.97 [0.22-17.72]	0.54	na.[b]	na.[b]	3.15 [0.36-27.8]	0.30
13q12.12	2.40 [0.48-12.1]	0.29	2.09 [0.25-17.3]	0.49	3.20 [0.65-15.8]	0.15	3.24 [0.59-17.7]	0.17
15q11.2	1.46 [1.02-2.07]	0.038	1.25 [0.86-1.83]	0.24	1.90 [1.29-2.79]	0.0010	0.91 [0.53-1.55]	0.72
PWAS	na.[b]	na.[b]	46.5 [20.1-108]	3.5×10^{-19}	na.[b]	na.[b]	15.1 [7.38-30.9]	1.1×10^{-13}
15q13.3	10.4 [5.03-21.5]	2.7×10^{-10}	2.28 [1.04-5.00]	0.041	7.33 [3.32-16.2]	8.1×10^{-7}	1.70 [0.62-4.62]	0.30
16p13.11	6.12 [3.63-10.3]	1.1×10^{-11}	2.04 [1.24-3.35]	0.0051	10.0 [6.60-15.2]	3.2×10^{-27}	0.50 [0.16-1.56]	0.23
16p12.1	1.49 [0.75-2.96]	0.25	1.24 [0.36-4.26]	0.73	1.64 [0.76-3.56]	0.21	0.63 [0.09-4.65]	0.65
16p11.2d	2.76 [1.07-7.11]	0.036	1.86 [0.69-5.01]	0.22	5.26 [2.17-12.8]	2.4×10^{-4}	1.20 [0.28-5.07]	0.81
16p11.2	5.14 [2.67-9.91]	1.0×10^{-6}	4.43 [2.76-7.09]	5.9×10^{-10}	5.47 [2.59-11.6]	8.7×10^{-6}	2.35 [1.20-4.62]	0.013
17p12	0.33 [0.04-2.49]	0.28	2.80 [0.88-8.86]	0.080	na.[b]	na.[b]	3.35 [1.00-11.2]	0.050
17q12	16.1 [5.90-43.8]	5.6×10^{-8}	2.96 [1.06-8.25]	0.038	na.[b]	na.[b]	2.01 [0.48-8.45]	0.34
22q11.2	18.5 [7.98-42.7]	9.6×10^{-12}	3.80 [2.15-6.71]	4.4×10^{-6}	11.4 [5.36-24.4]	2.8×10^{-10}	1.97 [0.85-4.60]	0.12
22q11.2b	1.66 [0.20-13.8]	0.64	na.[b]	na.[b]	6.38 [2.03-20.1]	0.0015	1.48 [0.20-11.2]	0.70

[a]rCNV-associated hazard ratios (HR) and 95% confidence intervals (CI95%) were derived for intellectual disability and epilepsy using a sex-stratified Cox proportional hazards (CPH) model with inverse probability of sampling (IPS) weights. [b]Comparisons involving <2 case carriers, or the CPH model failing a test of proportionality of hazards, were excluded.

© 2024 Vaez M et al. JAMA Psychiatry.

eReferences

1. Bybjerg-Grauholm, J., Pedersen, C.B., Bækvad-Hansen, M. *et al*. The iPSYCH2015 Case-Cohort sample: updated directions for unravelling genetic and environmental architectures of severe mental disorders. Preprint at https://www.medrxiv.org/content/10.1101/2020.11.30.20237768v1
2. Pedersen, C.B. The Danish Civil Registration System. *Scand J Public Health 2011;* 39: 22–25.
3. Mors, O., Perto, G.P., Mortensen, P.B. The Danish Psychiatric Central Research Register. *Scand J Public Health* 2011; 39: 54–57.
4. Schmidt, M., Schmidt, S.A.J., Sandegaard, J.L., Ehrenstein, V., Pedersen, L., Sørensen, H.T. The Danish National Patient Registry: a review of content, data quality, and research potential. *Clin Epidemiol* 2015; 7: 449–490.
5. Hollegaard, M.V., Grove, J., Grauholm, J. *et al*. Robustness of genomewide scanning using archived dried blood spot samples as a DNA source. *BMC Genet* 2011; 12: 58.
6. Pedersen, C.B., Bybjerg-Grauholm, J., Pedersen, M.G. et al. The iPSYCH2012 case–cohort sample: new directions for unravelling genetic and environmental architectures of severe mental disorders. Mol Psychiatry 2018; 23: 6-14.
7. Crawford, K., Bracher-Smith, M., Owen, D. et al. Medical consequences of pathogenic CNVs in adults: analysis of the UK Biobank. J Med Genet 2019; 56: 131-138.
8. Rehm, H.L., Berg, J.S., Brooks, L.D. *et al*. ClinGen--the Clinical Genome Resource. *N Engl J Med* 2015; 372: 2235-2242.
9. Riggs, E.R., Nelson, T., Merz, A. *et al*. Copy number variant discrepancy resolution using the ClinGen dosage sensitivity map results in updated clinical interpretations in ClinVar. *Hum Mutat* 2018; 39: 1650-1659.
10. Wang, K., Li, M., Hadley, D. *et al*.PennCNV: an integrated hidden Markov model designed for high-resolution copy number variation detection in whole-genome SNP genotyping data. *Genome Res 2007;* 17: 1665-1674.
11. McCarthy, S., Das, S., Kretzschmar, W. *et al*. A reference panel of 64,976 haplotypes for genotype imputation. *Nat Genet* 2016; 48: 1279-1283.
12. Montalbano, S., Sánchez, X.C., Vaez, M., Helenius, D., Werge, T., Ingason, A. Accurate and Effective Detection of Recurrent Copy Number Variants in Large SNP Genotype Datasets. *Curr Protoc* 2022; 2: e621.
13. Lumley T (2023). survey: analysis of complex survey samples. R package version 4.2.
14. Therneau T (2023). A Package for Survival Analysis in R. R package version 3.5-5.
15. Barlow, W.E., Ichikawa, L., Rosner, D., Izumi, S. Analysis of case-cohort designs. *J Clin Epidemiol 1999;* 52: 1165-1172.
16. Vanegas, L., Rondón, L., Paula, G. (2023). glmtoolbox: Set of Tools to Data Analysis using Generalized Linear Models. R package version 0.1.7.
17. Karczewski, K.J., Francioli, L.C., Tiao, G., et al. The mutational constraint spectrum quantified from variation in 141,456 humans. *Nature* 2020; 581: 434-443.
18. R Core Team (2022). R: A language and environment for statistical computing. R Foundation for Statistical Computing, Vienna, Austria
19. Malhotra, D., Sebat, J. CNVs: harbingers of a rare variant revolution in psychiatric genetics. *Cell* 2012; 148: 1223-1241.
20. Gudmundsson O.O., Walters G.B., Ingason A. *et al*. Attention-deficit hyperactivity disorder shares copy number variant risk with schizophrenia and autism spectrum disorder. *Transl* Psychiatry 2019; 9: 258.
21. Kendall K.M., Rees E., Bracher-Smith M. *et al*. Association of rare copy number variants with risk of depression. *JAMA Psychiatry* 2019; 76: 818–825.
22. Marshall C.R., Howrigan D.P., Merico D. *et al*. Contribution of copy number variants to schizophrenia from a genome-wide study of 41,321 subjects. *Nat Genet* 2017; 49: 27–35.
23. Rees E., Kendall K., Pardiñas A.F. *et al*. Analysis of intellectual disability copy number variants for association with schizophrenia. *JAMA Psychiatry* 2016; 73: 963–969.
24. Rees, E., Walters, J.T., Georgieva, L. *et al*. Analysis of copy number variations at 15 schizophrenia-associated loci. *Br J Psychiatry* 2014; 204: 108-114.

© 2024 Vaez M et al. JAMA Psychiatry.
