# Supplementary material for: Population-Based Risk of Psychiatric Disorders Associated With Recurrent Copy Number Variants
Source: JAMA Psychiatry. 2024 Jun 26;81(10):957–66. doi: 10.1001/jamapsychiatry.2024.1453 (PMC11209205; doi:10.1001/jamapsychiatry.2024.1453)
Supplement: Supplement 3. — Nonauthor collaborators [file jamapsychiatry-e241453-s003.pdf]

Supplemental Online Content: Nonauthor Collaborators
*First name, last name, and suffix (if applicable) are required and will appear in PubMed.

*Group Name(s): iPSYCH Investigators							
***First Name and Middle Initial(s)**	***Last Name**	***Suffix (eg, Jr, III)**	Academic Degrees	Institution	Location (city, state/province, country)	Role or Contribution, eg, chair, principal investigator	Group (if more than 1 Group listed in the byline) and/or Subgroup (eg, Steering Committee)
Anders D.	Børglum			Aarhus University	Aarhus, Denmark		
David M.	Hougaard			Statens Serum Institute	Copenhagen, Denmark		
Merete	Nordentoft			Mental Health Centre Copenhagen	Copenhagen, Denmark		
Ole	Mors			Aarhus University Hospital-Psychiatry	Aarhus, Denmark		
Preben B.	Mortensen			National Centre for Register-Based Research	Aarhus, Denmark		
Thomas	Werge			Institute of Biological Psychiatry	Roskilde, Denmark		
Jakob	Grove			Aarhus University	Aarhus, Denmark		
Thomas D.	Als			Aarhus University	Aarhus, Denmark		
Alfonso	Buil			Institute of Biological Psychiatry	Roskilde, Denmark		
Anders	Rosengren			Institute of Biological Psychiatry	Roskilde, Denmark		
Andrés	Ingason			Institute of Biological Psychiatry	Roskilde, Denmark		
Andrew J.	Schork			Institute of Biological Psychiatry	Roskilde, Denmark		
Dorte	Helenius			Institute of Biological Psychiatry	Roskilde, Denmark		
Jesper	Gådin			Institute of Biological Psychiatry	Roskilde, Denmark		
Richard	Zetterberg			Institute of Biological Psychiatry	Roskilde, Denmark		
Vivek	Appadurai			Institute of Biological Psychiatry	Roskilde, Denmark		
Joeri	Meijsen			Institute of Biological Psychiatry	Roskilde, Denmark		
Kajsa-Lotta	Georgii Hellberg			Institute of Biological Psychiatry	Roskilde, Denmark		
Bjarni J.	Vilhjálmsson			National Centre for Register-Based Research	Aarhus, Denmark		
Carsten B.	Pedersen			National Centre for Register-Based Research	Aarhus, Denmark		
Esben	Agerbo			National Centre for Register-Based Research	Aarhus, Denmark		
Jakob	Christensen			National Centre for Register-Based Research	Aarhus, Denmark		
Liselotte V.	Petersen			National Centre for Register-Based Research	Aarhus, Denmark		
Marianne	Gjørtz Pedersen			National Centre for Register-Based Research	Aarhus, Denmark		
Jonas	Byberg-Grauholm			Statens Serum Institute	Copenhagen, Denmark		
Marie	Bækvad-Hansen			Statens Serum Institute	Copenhagen, Denmark		
