# Supplementary material for: Population-Based Risk of Psychiatric Disorders Associated With Recurrent Copy Number Variants
Source: JAMA Psychiatry. 2024 Jun 26;81(10):957–66. doi: 10.1001/jamapsychiatry.2024.1453 (PMC11209205; doi:10.1001/jamapsychiatry.2024.1453)
Supplement: Supplement 4. — Data sharing statement [file jamapsychiatry-e241453-s004.pdf]

Data Sharing Statement

Vaez. Population-Based Risk of Psychiatric Disorders Associated With Recurrent Copy Number Variants. *JAMA Psychiatry*. Published June 26, 2024.
doi:10.1001/jamapsychiatry.2024.1453

Data
Data available: No

Additional Information
Explanation for why data not available: Regarding access to study data (other than sensitive person-level data, which by requirement of the data custodian and Danish legislation cannot be shared) please contact the corresponding author.
